# Development of Bispecific Antibody Targeting Human IL-17A and IL-6

**DOI:** 10.3390/antib15020029

**Published:** 2026-03-30

**Authors:** Beata Pamuła, Martyna Banach, Marta Mikońska, Karolina Korytkowska, Krzysztof Lacek, Oliwia Śniadała, Małgorzata Marczak, Krzysztof Flis, Aleksandra Sowińska, Damian Kołakowski, Jerzy Pieczykolan, Beata Zygmunt, Maciej Wieczorek, Olga Abramczyk

**Affiliations:** 1 Preclinical Development Department, Celon Pharma S.A., Ul. Marymoncka 15, 05-152 Kazuń Nowy, Polandmarta.mikonska@celonpharma.com (M.M.); krzysztof.lacek@celonpharma.com (K.L.); malgorzata.marczak@celonpharma.com (M.M.); krzysztof.flis@celonpharma.com (K.F.); aleksandra.sowinska@celonpharma.com (A.S.); damian.kolakowski@celonpharma.com (D.K.); maciej.wieczorek@celonpharma.com (M.W.); 2 Department of Cell Cultures and Genomic Analysis, Faculty of Medicine, Medical University of Lodz, Ul. Żeligowskiego 7/9, 90-752 Łódź, Poland

**Keywords:** bispecific antibody (BsAb), IL-6, IL-17A, VHH, nanobodies, cytokines, inflammatory diseases

## Abstract

**Background/Objectives**: Antibodies are a rapidly expanding field in drug discovery, but their monospecificity limits therapeutic applications, particularly in complex inflammatory diseases. Multispecific therapeutics, which combine variable regions targeting two or more antigens, offer potential advantages such as enhanced efficacy, broader target modulation, and reduced side effects. This study aimed to identify and characterize bispecific, VHH-based antibodies simultaneously targeting IL-6 and IL-17A—two key cytokines involved in autoimmune and chronic inflammatory conditions. **Methods**: A phage display screening was conducted using llama-derived VHH libraries to select binders against human IL-6 and IL-17A. Binding affinities of individual VHHs and assembled bispecific constructs were assessed using Bio-Layer Interferometry (BLI). Functional activity was evaluated using reporter cell lines responsive to IL-6 and IL-17A signaling. Biophysical and quality assessments of selected VHHs and bispecific antibodies were performed using the Uncle screening platform and LabChip capillary electrophoresis. **Results**: Several high-affinity VHH binders were identified for both IL-6 and IL-17A, and incorporated into bispecific antibody formats. The bispecific candidates exhibited simultaneous inhibition of both cytokine pathways in functional reporter assays. Biophysical characterization confirmed good stability and purity profiles for selected molecules. **Conclusions**: This study demonstrates the feasibility of generating stable, functional bispecific VHH-based antibodies targeting IL-6 and IL-17A. These constructs show potential as therapeutic agents for treating autoimmune and chronic inflammatory diseases by modulating multiple signaling pathways simultaneously.

## 1. Introduction

Diseases associated with impaired immune system function affect approximately 3–5% of the global population, significantly reducing patients’ quality of life. Despite advances in diagnostics and therapies, the underlying etiological mechanisms remain poorly understood [1,2]. To date, around 150 distinct autoimmune diseases have been identified. The diversity and rising incidence of these disorders present major challenges for clinicians, researchers, and healthcare systems, which must develop cost-effective strategies for diagnosis, treatment, and prevention [3]. In Europe, total direct healthcare costs for autoimmune diseases are estimated at €57.2 billion, with drug expenditures accounting for €12.0 billion (21% of total costs), underscoring the substantial economic burden of these conditions [4].

Cytokines, a diverse group of soluble proteins and glycoproteins, are produced by many immune cell types and play essential roles in regulating of cell proliferation, differentiation and their functionality. Inflammation, a complex immune response triggered by factors such as tissue damage or infection, is primarily regulated by pro-inflammatory cytokines and chemokines. Aberrant regulation of these signaling molecules contributes to the development of numerous immune-mediated diseases, including chronic inflammatory conditions, and cancer. Dysregulated cytokine expression is a hallmark of chronic inflammation, and plays a central role in disease pathogenesis, highlighting the therapeutic relevance of these molecules [5]. Accordingly, monitoring and modulating cytokine levels is of critical importance. Monoclonal antibodies (mAbs) targeting specific interleukins have already led to significant advances in clinical practice, and cytokines remain promising targets for therapeutic intervention [5].

Interleukin-6 (IL-6) is a 26 kDa pleiotropic cytokine with both pro- and anti-inflammatory functions. IL-6 exerts its biological effects through binding to the IL-6 receptor (IL-6R), followed by an association with the gp130 signaling subunit, activating JAK/STAT and Ras/MAPK pathways. This signaling pathway occurs via classical (cis-) or trans-signaling modes, involving membrane-bound or soluble IL-6R, respectively. IL-6 is secreted by various immune cells, including monocytes and macrophages, and is crucial in B and T cell activation, as well as for the differentiation of naïve CD4^+^ T cells into Th17 cells [6].

Chronic elevation in IL-6 levels has been linked to the pathogenesis of multiple inflammatory disorders. Consequently, therapeutic strategies targeting the IL-6/IL-6R axis have been developed. Tocilizumab, a humanized monoclonal antibody that blocks IL-6R, has been approved for conditions such as rheumatoid arthritis, Castleman’s disease, and COVID-19-related cytokine release syndrome [6]. Other IL-6-targeted therapies include Siltuximab and Olokizumab, both of which neutralize IL-6 [7,8].

Another key player in inflammation is interleukin-17A (IL-17A), a 35 kDa cytokine that forms homodimers and heterodimers (particularly with IL-17F) and is secreted primarily by Th17 cells [9]. IL-17A signals through a receptor complex composed of IL-17RA and IL-17RC, inducing multiple inflammatory and immune responses, including activation of key pathways such as NF-κB and MAPK, and promoting the expression of numerous target genes by modulating mRNA stability [10,11]. IL-17A is abundant in the synovial fluid and serum of patients with autoimmune diseases, such as psoriatic arthritis, and induces the production of pro-inflammatory cytokines (e.g., IL-6), chemokines (e.g., CXCL8), matrix metalloproteinases, and antimicrobial peptides [12].

Therapies that neutralize IL-17A or its receptor (IL-17RA) have been successfully applied to treat autoimmune disorders such as psoriasis and ankylosing spondylitis. Notably, Secukinumab, an IgG1/kappa antibody against IL-17A, and Ixekizumab, an IgG4 anti-IL-17A antibody, prevent the cytokine’s interaction with its receptor, and are approved for clinical use [13,14].

Despite the clinical success of monospecific antibodies, complex diseases involving multiple cytokine-driven pathways may benefit from more comprehensive targeting strategies. Bispecific antibodies capable of simultaneously neutralizing IL-6 and IL-17A represent a promising therapeutic approach to overcome the limitations of monospecific treatments. Such dual-action biologics may provide enhanced efficacy by disrupting distinct yet complementary inflammatory pathways involved in chronic immune-mediated diseases [5,15]. Some patients with rheumatoid arthritis do not respond adequately to conventional DMARDs and/or TNF inhibitors [16]. Similarly, in psoriasis, a proportion of patients fail to achieve sufficient clinical benefit from Secukinumab or Ixekizumab treatment [17,18]. The possible synergistic effect of bispecific antibody (BsAb) usage may justify the administration of lower Ab doses than those required for combinations of monospecific antibodies, which may result in potentially fewer and/or less intense side effects, which will be beneficial for patients [5,15,19,20].

In this study, we describe the development and evaluation of bispecific antibody candidates targeting IL-6 and IL-17A. We employed a phage display library encoding llama single-domain antibodies (VHHs) to isolate high-affinity binders. The binding properties of nanobodies and bispecific constructs were characterized using bio-layer interferometry (BLI). Functional activities were assessed in reporter cell assays, and biophysical properties were evaluated using the Uncle platform and LabChip capillary electrophoresis. These data support the potential of bispecific antibodies in modulating multifactorial cytokine signaling in immune-mediated pathologies.

## 2. Materials and Methods

### 2.1. Generation of the Immune Library

A single llama (Yumab GmbH, Braunschweig, Germany) was subcutaneously immunized with a cocktail of recombinant human antigens, IL-6 (30-212 aa) and IL-17A (24-155 aa) to generate a VHH (nanobody) library. Primary immunization was carried out on day 0 using complete Freund’s adjuvant (CFA; Sigma-Aldrich, Burlington, MA, USA). Booster immunizations containing 0.5 mg of each protein in incomplete Freund’s adjuvant (IFA; Sigma-Aldrich, Burlington, MA, USA) were administered on days 28, 42, and 56. On day 60, peripheral blood was collected for lymphocyte isolation and total RNA was extracted. First-strand cDNA was synthesized using reverse transcriptase and used as a template for amplification of the VHH-coding sequences by PCR. Amplified VHH fragments were cloned into a phagemid vector encoding a pelB signal sequence for periplasmic expression, c-Myc and 6×His·tag for protein detection and purification purposes, and pIII coat protein of M13 phage as a carrier protein. The resulting constructs were transformed into *Escherichia coli* (*E. coli*), and the library size was estimated based on the number of colony-forming units (cfu), reaching 1.7 × 10^8^ cfu. Insert presence and reading-frame accuracy were confirmed by PCR and sequence analysis. The presence of VHHs on phage particles was verified by immunoblotting using anti-Myc or anti-His·tag antibodies.

### 2.2. Selection of VHHs

Nanobodies binding to IL-6 or IL-17A were selected from the phage display library through three rounds of selection using antigens immobilized on a plate or soluble biotinylated antigens with magnetic beads (0.5–5 μg of antigen). Antigen biotinylation was conducted using the EZ-Link™ NHS-PEG4-Biotinylation Kit (Thermo Fisher Scientific Inc., Waltham, MA, USA) following the manufacturer’s instructions, and confirmed by Western blot with an anti-biotin-HRP antibody (Abcam Ltd., Cambridge, UK). After blocking with MPBST (2% milk in 1× PBST), the phage library was incubated with the antigens for 2 h at room temperature (RT), followed by washing with 1× PBST buffer (1× PBS, 0.05% Tween20). Bound phages were eluted using a trypsin solution (10 μg/mL) in PBS, and the eluted phages were used to infect *E. coli* TG1 strain (OD_600_ = 0.5) in fresh 2× YT medium, supplemented with glucose (100 mM) and ampicillin (100 µg/mL), at 37 °C for 1 h. Polyclonal phage populations were amplified by co-infecting bacteria with the M13KO7 helper phage (New England Biolabs (NEB), Ipswich, MA, USA) for 30 min at 37 °C without shaking, followed by 30 min at 37 °C with shaking, to generate recombinant phages displaying antibody fragments. After centrifugation at 4000× *g* for 30 min, the pellet was incubated overnight at 30 °C with shaking in fresh 2× YT medium containing ampicillin (100 µg/mL) and kanamycin (50 µg/mL). This antibiotic selection ensured the presence of M13KO7 during incubation, which increased the yield of phagemid ssDNA production. The supernatant was then used for the next round of panning. The final phage pool after the third round was measured by infecting TG1 bacteria with serial phage dilutions, followed by plating on solid 2× YT medium supplemented with glucose (100 mM) and ampicillin (100 μg/mL). Colonies were counted after overnight incubation at 37 °C.

### 2.3. Production of Soluble Antibody Fragments

Individual clones picked after the third round of selection were transferred to 2× YT medium supplemented with glucose (100 mM) and ampicillin (100 μg/mL) in a microtiter plate and incubated overnight at 37 °C. The overnight cultures were then diluted 20× in fresh 2× YT medium supplemented with glucose and ampicillin and incubated for 2 h at 37 °C with shaking. After centrifugation at 4000× *g* for 10 min, the supernatants were removed and the pellets were resuspended in 2× YT medium supplemented with potassium phosphate buffer (10%; *v*/*v*), sucrose (50 mM), ampicillin (100 μg/mL), and IPTG (50 μM) to induce protein production. Following overnight incubation at 30 °C, the bacteria were centrifuged, and the supernatants containing soluble proteins were retained for ELISA and BLI screening.

### 2.4. ELISA Screening of VHHs

Plates were coated with IL-6 or IL-17A (100 ng per well; homemade; no-Taq) by overnight incubation at 4 °C. After incubation, the plates were blocked with MPBST (5% milk powder in 1× PBST) for 1 h at room temperature to prevent non-specific binding, and then washed three times with 1× PBST (1× PBS, 0.05% Tween20). The plates were incubated for 1 h at room temperature with the culture medium containing phage-displayed VHHs after expression in TG1 bacteria, and then washed three times with 1× PBST to remove unbound phages. The plates were then incubated with an anti-c-myc antibody (1:5000 in MPBST) for 1 h at room temperature to detect c-myc-tagged VHH-containing fusion proteins, followed by three washes with 1× PBST. The signal was developed by adding TMB (Sigma, St. Louis, MO, USA), an HRP substrate, to each well and incubating for 10 min at room temperature. The reaction was stopped by adding 1 M HCl to each well, and absorbance was measured at 450 nm using a spectrophotometer (Multiskan; Thermo Fisher Scientific Inc.) to quantify the binding of VHHs to the antigens.

### 2.5. Off-Rate Screening of VHHs

Off-rate screening of VHHs was performed directly in the culture medium in a 96-well black plate (GREINER via Sigma-Aldrich, Poznań, Poland) on an Octet RED96 instrument (Sartorius AG, Göttingen, Germany) at 25 °C with agitation at 1000 rpm. The medium fraction of the VHH anti-IL-17A and anti-IL-6 selected positive clones after primary screening (ELISA) were transferred to a new 96-well plate for secondary screening. Homemade Fc-tagged target proteins, IL-6 or IL-17A, at 10 µg/mL concentration in 1× Kinetic Buffer (Sartorius AG) were immobilized on anti-human IgG Fc capture biosensors (AHC; Sartorius AG) for 5 min. After loading, the sensors were washed with 1× Kinetic Buffer for 1 min, blocked for 5 min with *E. coli* (TG1 strain) mock medium containing no VHH protein, transferred to the wells containing the VHH protein for the association step (5 min), and transferred back to the mock medium for the dissociation step (5 min). Dissociation rate constants (k_off_) for each protein were calculated using a 1:1 interaction model (fitting local) with the ForteBio data analysis software HT 10.0.

### 2.6. VHHs Expression and Purification

Phagemids encoding VHH with His·tag on the C terminus were isolated from the TG1 *E. coli* strain using MagMAX kit (Applied Biosystems, Thermo Fisher Scientific Inc., Waltham, MA, USA), following the manufacturer’s protocol. Each construct was transformed back into *E. coli* strain BL21(DE3) (NEB). Transformed bacteria were grown in LB broth at 37 °C until the OD_600_ reached 0.8, and then cooled to 30 °C. Expression of recombinant proteins was induced by adding 0.5 mM IPTG (BLIRT S.A., Gdańsk, Poland), and the bacterial cultures were incubated for 16 h at 30 °C. Cells were harvested by centrifugation (15 min, 12,000× *g*, 4 °C). Supernatant (culture medium) was collected, while the cell pellets were resuspended in 100 mM Tris pH 8.0, 20% sucrose, and 0.25 mM EDTA, and incubated for 20 min with gentle rocking at 4 °C to isolate periplasmic fractions. After 20 min of incubation, soluble fraction was clarified by centrifugation (15 min, 15,000× *g*, 4 °C); supernatant was saved for further steps, and the cell pellets were resuspended in a second buffer containing 5 mM MgSO_4_. The cells were incubated for 20 min with gentle rocking at 4 °C, sedimented by centrifugation (15 min, 15,000× *g*, 4 °C), and a second soluble fraction was collected. Both resulting periplasmic fractions were pooled with the medium fraction. Each sample (250 mL) was filtered through a 0.22 µm filter (MERCK, Darmstadt, Germany) and mixed with 1 mL (250 µL of settled beads) of Ni-NTA magnetic beads (Thermo Fisher Scientific Inc.) equilibrated with 50 mM phosphate buffer, pH 7.0, 300 mM NaCl, and 0.05% Tween20. All samples were incubated for 1 h with gentle rocking at 4 °C. The magnetic beads were collected using magnetic stands, diluted in 5 mL of equilibration buffer (50 mM phosphate buffer, pH 7.0, 300 mM NaCl, 0.05% Tween20) and used for the next step of automative purification. Briefly, the 5 mL deep 24-well plates were used for protein purification using the KingFisher Flex Purification Automate (Thermo Fisher Scientific Inc.), following the manufacturer’s protocol. For each purification, the following buffers were used: equilibration buffer (50 mM phosphate buffer, pH 7, 300 mM NaCl, 0.05% Tween20), wash buffer (50 mM phosphate buffer, pH 7, 300 mM NaCl, 0.05% Tween20, 15 mM imidazole), and elution buffer (50 mM phosphate buffer, pH 7, 300 mM NaCl, 300 mM imidazole). The collected fractions were concentrated and the buffer was replaced with 50 mM phosphate buffer, pH 7.0, 300 mM NaCl, 5% glycerol using AMICON filtration units (10 MWCO; MERCK). Concentrations of purified proteins were determined by absorbance measurements at 280 nm using a NanoDrop spectrophotometer (IMPLEN GmbH, Munich, Germany).

### 2.7. Protein Thermal Stability

The thermal stability of nanobodies or a bispecific antibody was detected using Uncle, a multifunctional protein stability screening platform (Unchained Labs, Pleasanton, CA, USA) that integrates three distinct measurement modes—fluorescence, static light scattering (SLS), and dynamic light scattering (DLS). All samples were clarified by centrifugation at 10,000× *g* for 10 min and then immediately analyzed via Uncle at a concentration of 1 mg/mL (VHHs) and 10 mg/mL (bispecific antibody). Purified protein samples were heated from 25 °C to 95 °C at a rate of 0.5 °C/min, and the full spectrum fluorescence, SLS, and DLS data were recorded. The melting midpoint temperature (T_m_) was calculated by the Uncle Analysis software (Unchained Labs; version 6.01) for all VHHs. In addition, for the bispecific antibody, thermal-induced aggregation onset, and the polydispersity index (PDI) were determined based on SLS and DLS profiles.

### 2.8. Purity Determination

Purity analyses of VHHs or a bispecific antibody were performed by capillary electrophoresis on the LabChip GXII 24 Touch instrument (Revvity Inc., Waltham, MA, USA) using Protein Express Assay Reagent Kit and Protein Express Chip (Revvity Inc.). In addition, 1 µg of each VHH or 2.5 µg of bispecific antibody in 2 µL were mixed with 7 µL of sample buffer prepared by mixing 700 µL of HT Protein Express sample buffer with 24.5 µL of 2-mercaptoethanol (BME; for reducing condition assay). The samples were incubated at 95 °C for 5 min. The bispecific antibody was also prepared under non-reducing conditions (w/o BME). After cooling to room temperature, 35 µL of water was added to each sample before loading onto the instrument. The gel dye, ladder, and Protein Express Chip were prepared according to the manufacturer’s protocol (Revvity Inc.). In addition, the bispecific antibody was subjected to enzymatic deglycosylation using PNGase F enzyme according to the manufacturer’s protocol (NEB). Deglycosylation was performed under both reducing and non-reducing conditions, and the resulting samples were processed and analyzed as described above. Samples were analyzed using the HT Protein Express 100 script for VHHs and HT Protein Express 200 script for the bispecific antibody.

### 2.9. Affinity Determination of Purified VHHs

The binding affinities of selected VHHs to IL-6 and IL-17A were measured on an Octet RED96 instrument (Sartorius AG) at 25 °C, with agitation at 1000 rpm in a 96-well black plate (GREINER). IL-6 or IL-17A with FcTag (10 µg/mL; homemade) were immobilized on anti-human IgG Fc capture biosensors (AHC; Sartorius AG) for 2 min. Following a 1 min wash step in 1× Kinetic Buffer (Sartorius AG), the sensors were transferred to wells containing VHHs (31–500 nM) in Kinetic Buffer. After the association phase (2 min), the sensors were moved to wells containing only Kinetic Buffer (dissociation phase) for 2 min. The binding data were analyzed with the ForteBio data analysis software HT 10.0, and affinity constants (KD) were calculated using a 1:1 interaction model (fitting global).

### 2.10. Method of Assessment of VHHs’ Biological Activity Using HEK-Blue Reporter Cells

HEK-Blue IL-6 (#hkb-il6; InvivoGen, San Diego, CA, USA) or HEK-Blue IL-17 (#hkb-il17; InvivoGen) reporter cells were used to assess the biological activity of VHHs. Cells were stimulated with IL-6 (1 ng/mL; PeproTech Inc., Rocky Hill, NJ, USA) and IL-17A (10 ng/mL; ACROBiosystems, Newark, DE, USA) in the presence of various concentrations of each VHH, ranging from 0.3 nM to 600 nM for IL-6 and from 0.003 nM to 100 nM for IL-17A. The cytokines, IL-6 or IL-17A, were pre-incubated with each VHH for 15 min at 37 °C in a 5% CO_2_ atmosphere. Subsequently, 30,000–50,000 viable cells were added to each well and incubated for 24 h at 37 °C in a 5% CO_2_ atmosphere. IL-6 stimulation activated the STAT3-inducible SEAP-coding reporter gene, releasing SEAP into the medium. Similarly, IL-17A stimulation activated a signaling cascade involving NF-κB and AP-1, also inducing SEAP production. The resulting medium samples were then incubated with QUANTI-Blue solution (AP substrate; InvivoGen) for 30–60 min at 37 °C, and SEAP levels were measured using a spectrophotometer at 620 nm. All cell culture procedures were performed according to the manufacturer’s protocol. The IC_50_ values were determined using a three-parameter dose–response inhibition model (nonlinear fit) using GraphPad Prism ver. 10.

### 2.11. Design, Construction and Molecular Optimization of VHH-Based Bispecific Antibodies

The gene encoding the single-chain bispecific antibody, composed of an anti-IL-6 VHH (A5), an anti-IL-17A VHH (D3), and the Fc region of human IgG1, was codon-optimized for mammalian expression (Geneious Prime ver. 2023.0.4; GraphPad Software LLC, Boston, MA, USA) and synthesized by GenScript Biotech (Nanjing, China). The construct was cloned into the pcDNA3.1 expression vector. The two VHH domains were genetically linked in a head-to-tail orientation via a flexible (G_4_S)_3_ linker and fused to the Fc fragment to enable dimerization and prolong serum half-life (designated CPBT0853). To modulate effector functions and reduce Fc-mediated immune activation, the IgG1 Fc region was replaced with the Fc domain of human IgG4. The resulting construct retained the same VHH configuration and linker architecture as the IgG1-based molecule and was designated CPBT1174. To further reduce potential immunogenicity, particular amino acid residues in the frameworks of the VHH domains A5 and D3 were selected using several web servers: IGBLAST [https://www.ncbi.nlm.nih.gov/igblast/igblast.cgi] (accessed on 22 January 2026)—alignment to closest human germline sequences; ABYSIS [http://www.abysis.org/abysis/sequence_input/key_annotation/key_annotation.cgi] (accessed on 22 January 2026)—identification of unusual residues in humans and their percentage; DDMut [https://biosig.lab.uq.edu.au/ddmut/submit_prediction] (accessed on 22 January 2026)—determination of the effects of mutations on protein stability. Then, the framework residues were humanized, based on the results from the above web servers with human VH germline families, while preserving the complementarity-determining regions (CDRs) to maintain antigen specificity and binding affinity. This final humanized bispecific construct was designated CPBT1269. For experimental control purposes in the assay described in Section 2.17, two monospecific antibodies targeting IL-6 (CPBT1777, based on the A5 VHH clone) or IL-17A (CPBT1776, based on the D3 VHH clone) were also generated. These constructs consisted of the respective VHH domains fused to the Fc region of human IgG1 and were produced using an analogous expression and purification strategy.

### 2.12. Expression and Purification of Bispecific Antibodies

All plasmids based on the pcDNA3.1 (+) vector backbone for transient expression were prepared using the Endotoxin-Free MaxiPrep kit (Macherey-Nagel GmbH & Co. KG, Düren, Germany), following the manufacturer’s instructions. A bispecific antibody was produced in transiently transfected ExpiCHO-S cells (Thermo Fisher Scientific Inc.) using the FectoCHO transfection kit (Polyplus-transfection SA, Illkirch, France) according to the manufacturer’s protocol. The culture medium was harvested 6 days post-transfection, or when the cell viability dropped below 70%, and clarified by centrifugation (30 min, 7000× *g*, RT). The medium was additionally filtered using a 0.22 μm Bottle Top Filter (Sarstedt AG & Co. KG, Nümbrecht, Germany) before the subsequent purification process. The antibodies were purified by affinity chromatography using MabCaptureC resin (Thermo Fisher Scientific Inc.). For column equilibration and washing steps, 1× PBS pH 7.4 was used, whereas elution was performed using PBS with a linearly increasing gradient of 50 mM glycine pH 3.0. A final polishing step of the bispecific antibody was carried out by preparative size exclusion chromatography (HiLoad 16/600 Superdex 200 pg; Cytiva, Washington, DC, USA), during which the protein solution was exchanged to 1× PBS pH 6.8, 5% glycerol. Antibodies were concentrated using Amicon filters (50K MWCO; Sigma), and protein concentrations were determined by UV-VIS spectroscopy at 280 nm (NanoDrop; IMPLEN GmbH). Antibody purity was analyzed by SDS-PAGE (4–20% Mini-Protean TGX Precast gel; Bio-Rad Laboratories Inc., Hercules, CA, USA) and the LabChip GXII 24 Touch instrument using the HT Antibody Express 200 script.

### 2.13. Affinity Determination of Purified Bispecific Antibody CPBT0853

The binding of human, cynomolgus monkey (*Macaca fascicularis*), and mouse IL-6 and IL-17A (ACROBiosystems), human complex IL-17A/F (R&D System, Minneapolis, MN, USA) and human IL-17E and IL-17F (ACROBiosystems) to the anti-IL-6/anti-IL17A bispecific antibody CPBT0853 was analyzed using an Octet RED96 instrument (Sartorius AG). Dissociation constants (KD) were determined at seven concentrations for each cytokine, ranging from 0.78 nM to 100 nM, diluted in 1× Kinetic Buffer (Sartorius AG). All measurements were performed at 25 °C using a 96-well black plate (GREINER) agitated at 1000 rpm. The antibodies were immobilized on anti-human IgG Fc capture biosensors (AHC; Sartorius AG) at a concentration of 10 µg/mL for 2 min. After loading, the sensors were washed with 1× Kinetic Buffer for 1 min. The sensors were then transferred to wells containing IL-17A at varying concentrations for the association step (2 min) and subsequently transferred back to 1× Kinetic Buffer for the dissociation measurements (5 min). This process was followed by a 30 s wash in 1× Kinetic Buffer. The sensors were then transferred to wells containing IL-6 at different concentrations for the association step (2 min) and subsequently transferred back to 1× Kinetic Buffer for the dissociation measurements (5 min). For all other cytokines, including human IL-17E, IL-17F, and IL-17A/F complex, as well as murine and cynomolgus monkey IL-6 and IL-17A, binding to CPBT0853 was assessed using a standard single-analyte interaction format, where only one cytokine was associated per measurement, unlike the sequential dual-cytokine assay described above. All measurements were corrected for baseline drift by subtracting data from a control sensor exposed only to 1× Kinetic Buffer. Data were analyzed using a 1:1 interaction model (global fitting) with the ForteBio data analysis software HT 10.0. It should be noted that for very-high-affinity interactions (low picomolar range), kinetic parameters derived from BLI may represent apparent values due to avidity effects resulting from bivalent Fc-mediated immobilization and instrumental detection limits for very slow dissociation rates.

### 2.14. Measurement of the Potential of CPBT0853 to Inhibit IL-17A/IL-17RA and IL-6/IL-6R Interactions

To assess the ability of the bispecific antibody to inhibit the binding of cytokines to their receptors, the Octet RED96 instrument (Sartorius AG) was utilized. All measurements were performed at 25 °C using a 96-well black plate (GREINER) agitated at 1000 rpm. Biotinylated cytokines (ACROBiosystems) were immobilized on a streptavidin sensor (SA; Sartorius AG) at a concentration of 100 nM for 2 min. After loading, the sensors were washed with 1× Kinetic Buffer for 30 s. The sensors were then transferred to wells containing bispecific antibodies at varying concentrations (12.5–100 nM) for the association step (2 min) and subsequently transferred back to 1× Kinetic Buffer for the dissociation measurements (1 min). Next, the sensors were washed with 1× Kinetic Buffer for 30 s and transferred to wells containing IL-17AR or IL-6R at a concentration of 200 nM for 2 min, followed by transfer back to 1× Kinetic Buffer for the dissociation measurements (2 min). The thickness of the biological layer of the complex IL-17A with its receptor IL-17AR or IL-6 with its receptor IL-6R was analyzed using ForteBio data analysis software HT 10.0. All measurements were corrected for baseline drift by subtracting data from a control sensor exposed only to 1× Kinetic Buffer. The results were compared with those obtained using the monospecific antibody Ixekizumab (Taltz; Eli Lilly Nederland B.V., Utrecht, The Netherlands), an anti-IL-17A antibody used in the treatment of psoriasis; and Siltuximab (prepared in-house), an anti-IL-6 antibody used for treating Castleman disease.

### 2.15. Assessment of Neutralizing Activity of Bispecific Antibody Using HEK-Blue Reporter Cells

HEK-Blue IL-6 reporter cells (#hkb-il6; InvivoGen) or HEK-Blue IL-17 reporter cells (#hkb-il17; InvivoGen) were used to assess the activity of the bispecific antibody CPBT0853, respectively. The cells were stimulated with IL-6 (1 ng/mL; ACROBiosystems) or IL-17A (10 ng/mL; ACROBiosystems) and human complex IL-17A/F (10 ng/mL; R&D System) in the presence of various concentrations of the bispecific antibody ranging from 0.0003 nM to 100 nM. Prior to stimulation, the cytokines IL-6 or IL-17A were pre-incubated with the bispecific antibody for 15 min at 37 °C in a 5% CO_2_ atmosphere. Subsequently, either 30,000 or 50,000 viable cells were added to each well and incubated for 24 h at 37 °C in a 5% CO_2_ atmosphere. The IL-6 stimulation activated the STAT3-inducible SEAP-coding reporter gene, resulting in the release of the SEAP protein into the medium. Similarly, IL-17A stimulation triggered a signaling cascade involving NF-κB and AP-1, also leading to SEAP secretion. After incubation, the medium was mixed with QUANTI-Blue solution (InvivoGen) and incubated for 30–60 min at 37 °C. SEAP levels were then measured using a spectrophotometer at 620 nm. All cell culture procedures were performed according to the manufacturer’s protocol (InvivoGen). The IC_50_ values were calculated using a three-parameter dose–response inhibition model in GraphPad Prism software ver. 10.

### 2.16. Method for Measuring Inhibition of the IL-6-Induced STAT Signaling by the Bispecific Antibody

Peripheral blood mononuclear cells (PBMCs) from three different healthy donors from the Regionalne Centrum Krwiodawstwa i Krwiolecznictwa w Warszawie (Regional Centre of Blood Donation and Blood Treatment in Warsaw, Poland) were used to evaluate the activity of a bispecific antibody targeting IL-6 and IL-17A. PBMCs were isolated following the protocol described at https://www.reprocell.com/blog/biopta/pbmc-isolation-from-buffy-coat-samples (*Protocol for PBMC isolation from buffy coat samples*, REPROCELL Europe; Accessed on 22 January 2026). Prior to the assay, AIM V medium (Thermo Fisher Scientific) was warmed to 37 °C in a water bath, and 9 mL of medium was added to a 15 mL conical tube. PBMCs were thawed in a water bath, transferred to the conical tube containing AIM V medium, and centrifuged at 450× *g* for 5 min at room temperature. The supernatant was aspirated, and the cells were resuspended in 5 mL of fresh AIM V medium. Cells were seeded into 96-well plates (100 µL/well) in dilution 5 × 10^5^ cells per well in AIM V medium and incubated for 30 min at 37 °C in a 5% CO_2_ atmosphere before stimulation. For stimulation, PBMCs were incubated with IL-6 (1 ng/mL; ACROBiosystems) in the presence of various concentrations of the bispecific antibody CPBT0853, ranging from 0.003 nM to 100 nM. IL-6 was pre-incubated with the antibody for 20 min at room temperature. IL-6/antibody mixture was added to the well with PMBC and incubated for 30 min at 37 °C in a 5% CO_2_ atmosphere. IL-6 stimulation led to the activation of STAT3 and STAT1 pathways via phosphorylation. Following incubation, the cells were fixed and stained with anti-pSTAT1 (AF647) and anti-pSTAT3 (AF488) antibodies (BD Biosciences, Franklin Lakes, NJ, USA) to quantify phosphorylated STAT1 and STAT3. Flow cytometric analysis was performed using a FACS Symphony A1 (BD Biosciences). The percentage of pSTAT-positive lymphocytes was determined, and IC_50_ values were calculated using a three-parameter dose–response inhibition model (nonlinear fit) in GraphPad Prism software ver. 10.

### 2.17. Assessment of Biological Activity of Bispecific Antibodies in Human Fibroblast-like Synoviocytes (HFLS)

Human Fibroblast-Like Synoviocyte cells (HFLS; Cell Applications Inc. via Sigma, St. Louis, MO, USA) were used to assess the activity of a bispecific antibody targeting IL-6 and IL-17A. All cell culture procedures were conducted according to the supplier guidelines described at https://www.sigmaaldrich.com/PL/pl/technical-documents/protocol/cell-culture-and-cell-culture-analysis/primary-cell-culture/human-fibroblast-like-synoviocytes (Sigma; accessed on 22 January 2026). Cells were seeded into 12-well plates at a density of 7 × 10^5^ cells/mL (1 mL per well) and cultured overnight at 37 °C in a 5% CO_2_ atmosphere. The next morning, the medium was removed, and the cells were treated with 1 mL of test protein solutions prepared in growth medium. HFLS were stimulated with soluble IL-6 receptor (sIL-6R; 100 ng/mL, PeproTech) and recombinant human IL-17A (100 ng/mL, ACROBiosystems). HFLS cells lack expression of the membrane-bound IL-6 receptor (mIL-6R) but express gp130. Therefore, the addition of soluble IL-6R (sIL-6R) enables IL-6 trans-signaling through formation of the IL-6/sIL-6R complex, which binds to gp130 and induces a pro-inflammatory response. Before stimulation, these cytokines were pre-incubated with the bispecific antibody (1 nM) for 20 min at room temperature. Following pre-incubation, the HFLS cells were treated with the cytokine–antibody mixture and incubated for 24 h at 37 °C in a 5% CO_2_ atmosphere. After incubation, the supernatant was collected by centrifugation of culture medium at 10,000× *g* for 5 min. Cytokine levels in the supernatant were measured using the LEGENDplex™ Human Anti-Virus Response Panel (BioLegend, San Diego, CA, USA), following the manufacturer’s protocol. The results were compared with those obtained using reference antibodies: the monospecific antibody Ixekizumab (Taltz), an anti-IL-17A antibody used in the treatment of psoriasis, and Siltuximab (prepared in-house), an anti-IL-6 antibody used for treating Castleman disease. Monospecific formats of the bispecific antibody, i.e., anti-IL-6 (CPBT1777) and anti-IL-17A (CPBT1776), were used separately as an additional control.

## 3. Results

### 3.1. Identification of Lead VHH Candidates

#### 3.1.1. Generation of VHH Immunological Library and Selection

**Figure 1:**
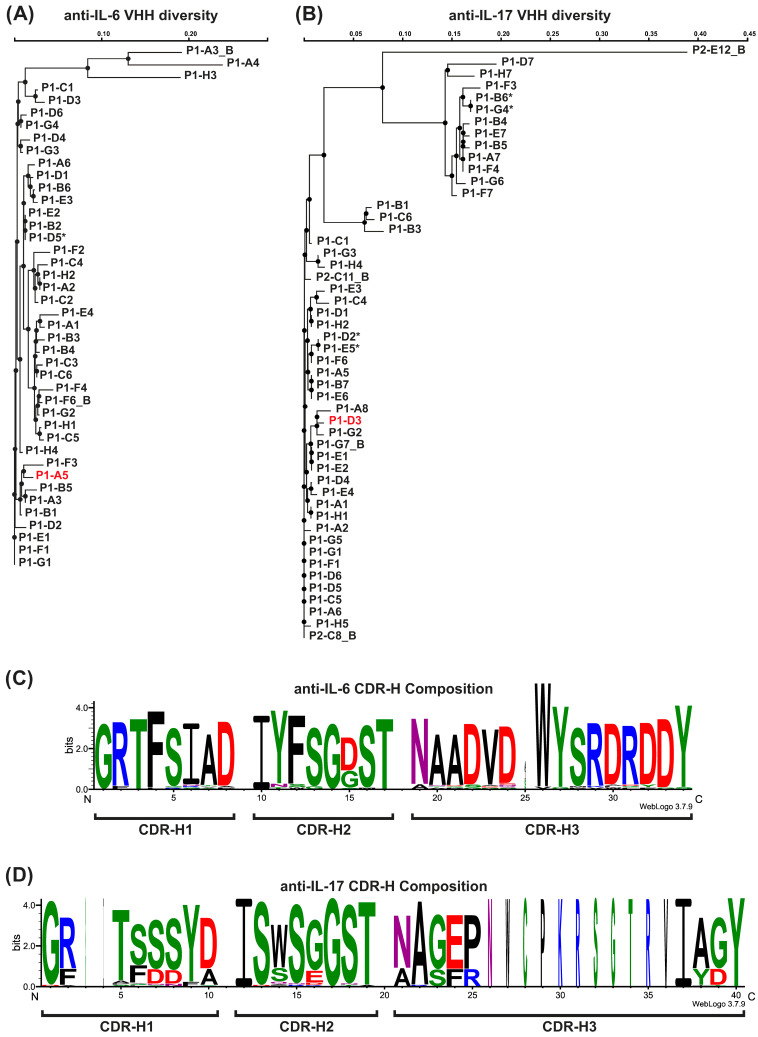
Characterization of VHH clone diversity. (**A**,**B**) Dendrograms resulting from multiple sequence alignment of amino acid sequences of VHH clones specific for IL-6 (**A**) and IL-17A (**B**), performed using Clustal Omega [https://www.ebi.ac.uk/jdispatcher/msa/clustalo] (EMBL-EBI service providing bioinformatics tools; Accessed on 22 January 2026) [21]. An asterisk (*) indicates clones that, at the phage library stage, contained a STOP codon in the coding sequence and required the presence of a corresponding suppressor tRNA provided by the *E. coli* host cell for protein synthesis. In red, two clones are marked that were selected at the end of the development stage for final BsAb assembly. (**C**,**D**) Residue conservation presented as a sequence logo representation of complementarity-determining regions’ (CDRs) repertoire among anti-IL-6 clones (**C**) and anti-IL-17A ones (**D**) obtained using WebLogo 3, a web-based application [https://weblogo.threeplusone.com/create.cgi] (accessed on 22 January 2026) [23,24]. The overall height of the stack at each position indicates the sequence conservation at that position, while the height of amino acid symbols within the stack represents the relative frequency of each amino acid. Moreover, the width of the stack is proportional to the fraction of the amino acid presence at a particular position, giving a richer picture of sequence conservation. The color scheme used shows amino acids according to their chemical properties.

Following the third round of biopanning in phage display, a total of 50 anti-IL-17A clones and 42 anti-IL-6 clones were selected using ELISA screening. After this initial selection, DNA was isolated from the bacterial clones and subjected to sequencing. Sequence alignment was performed using Clustal Omega (ClustalΩ) software to show sequence similarity and to identify and eliminate duplicate clones with identical sequences [21]. Since the conserved regions of VHH frameworks may overestimate the overall sequence similarity, the sequences of highly variable epitope-recognizing regions, i.e., CDRs, were separately analyzed and their multiple sequence alignment graphically presented as a sequence logo (Figure 1). The sequence similarity searches showed a high enrichment in VHH populations for both targets, with high similarity. Despite some clones showing only small differences in their frameworks, all identified unique clones were retained for further evaluation to explore the full range of potential functional variations. A schematic overview of the VHH discovery, screening, and lead selection workflow is provided in Supplementary Figure S1.

**Figure 2:**
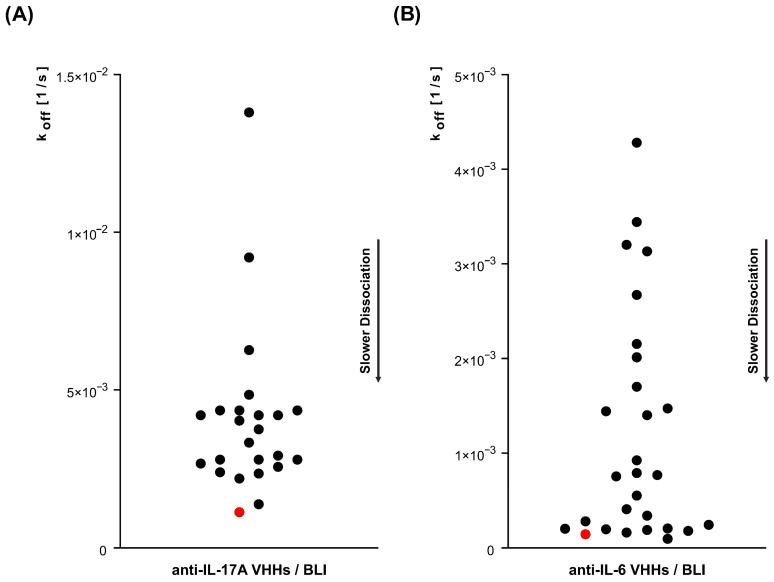
Dissociation rates (k_off_ in 1/s) of VHH clones specific for anti-IL-17A (**A**) and anti-IL-6 (**B**), were analyzed using bio-layer interferometry (BLI). The data illustrate the variability in dissociations rate across the selected clones for both IL-17A and IL-6. The clones highlighted in red, D3 for IL-17A and A5 for IL-6, represent the lead clones selected at a later stage of development.

The selected unique clones were further analyzed using bio-layer interferometry (BLI) to assess their binding affinities. During the initial screening stage, VHH clones were analyzed directly from bacterial supernatants, where antibody concentrations are variable and not precisely defined. Therefore, off-rate values (k_off_) were used as the primary comparative parameter, while full kinetic characterization (k_a_, k_off_, and KD) was performed only for purified VHHs at defined concentrations. This secondary screening step enabled the identification of 24 positive anti-IL-17A and 28 positive anti-IL-6 VHHs. Clones that did not exhibit binding affinity to the target were discarded. The results represent the dissociation rate (k_off_, in 1/s) of each clone, ranging from 2.86 × 10^−2^ to 1.38 × 10^−3^ for IL-17A (Figure 2A) and from 6.40 × 10^−3^ to 9.49 × 10^−5^ for IL-6 (Figure 2B). The k_off_ values for clones D3 and A5, which were selected as lead candidates in the later stages of the screening process, were 1.13 × 10^−3^ for IL-17A and 1.42 × 10^−4^ for IL-6. It is important to note that the ELISA signal obtained during primary screening may be influenced by the concentration of soluble proteins in the medium, which can vary significantly and is often unknown. Consequently, the ELISA signals may not accurately reflect the VHHs’ true affinities, necessitating k_off_ rate analysis in BLI for more precise affinity determination [22]. This systematic approach enabled the selection of diverse VHHs with the potential to generate effective bispecific antibodies targeting both IL-17A and IL-6, providing a robust foundation for subsequent development and characterization efforts.

#### 3.1.2. Production of VHHs

**Figure 3:**
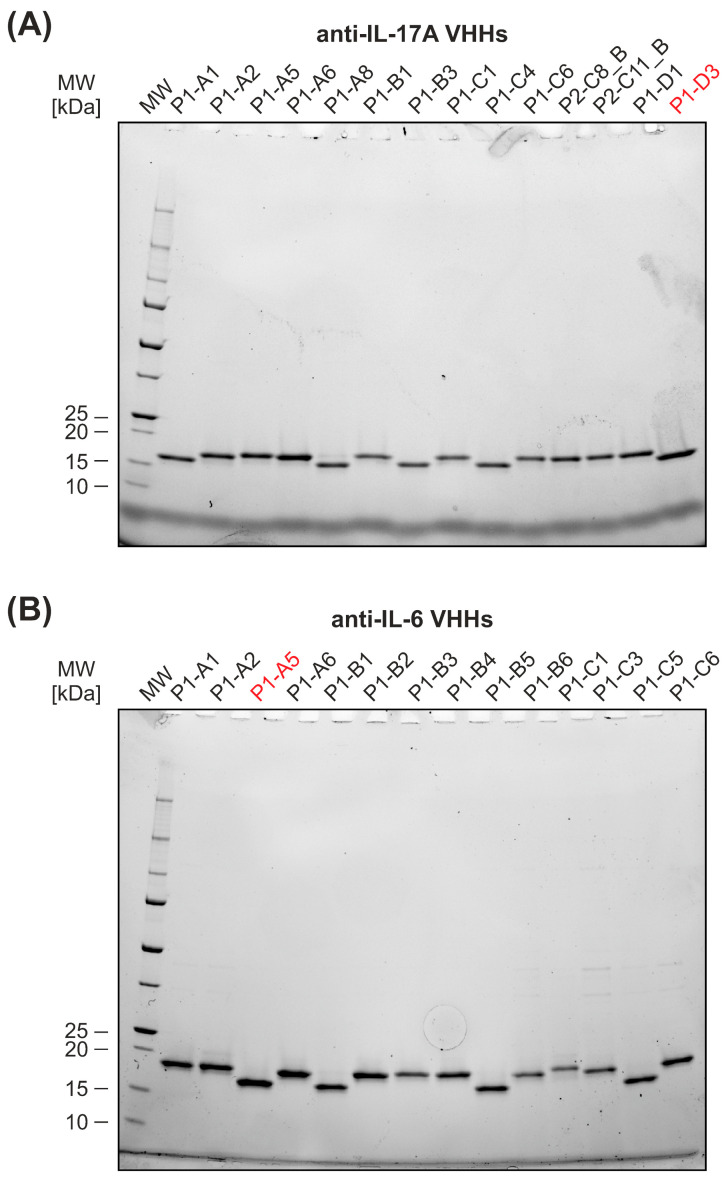
SDS-PAGE analysis of VHH clones under reducing conditions after purification using the KingFisher Flex station. Protein samples were loaded at 1 µg per well. Representative SDS-PAGE profiles of anti-IL-17A clones (**A**) and anti-IL-6 clones (**B**) illustrate the purity and integrity of the purified clones. The most promising VHHs, D3 for IL-17A and A5 for IL-6, are marked in red.

The purification of VHHs for screening purpose was performed using the KingFisher Flex station and Ni-NTA magnetic beads, which allowed us to obtain sufficient purity of the VHHs and significantly shortened the stage of preparation. The purified samples were subsequently concentrated and dialyzed, and protein concentrations were measured using a NanoDrop spectrophotometer. The purity of the produced VHHs was initially assessed using SDS-PAGE analysis (Figure 3).

**Figure 4:**
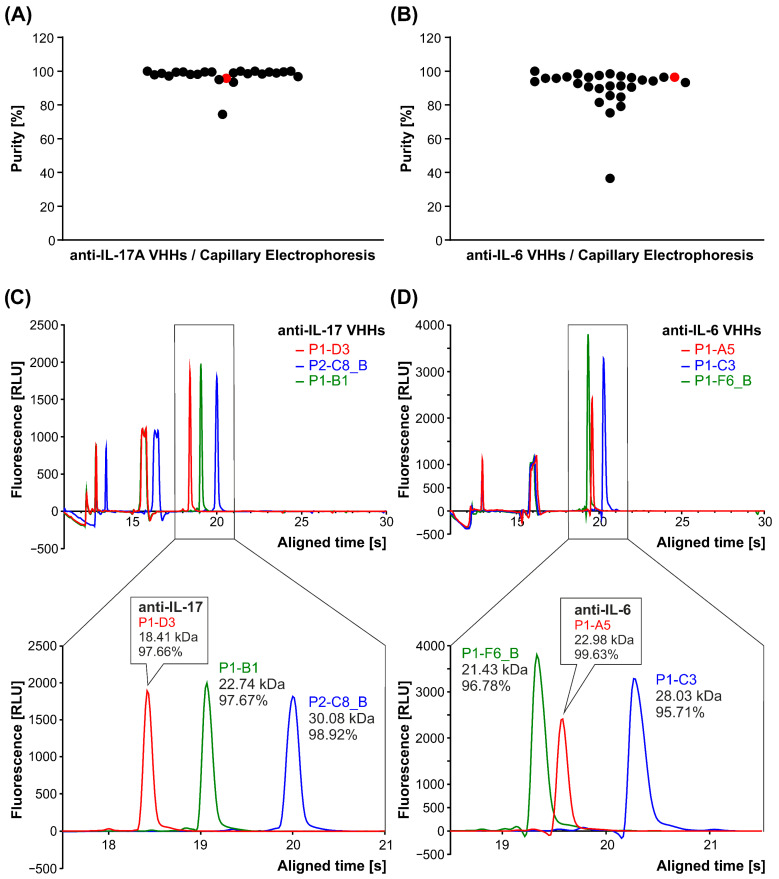
Purity analysis for all purified candidates from among anti-IL-17A clones (**A**) and anti-IL-6 clones (**B**) using capillary electrophoresis (LabChip system). The most promising VHHs, D3 for IL-17A and A5 for IL-6, are marked in red. Exemplary overlapped data analysis results obtained from the capillary electrophoresis are shown for anti-IL-17A clones (**C**) and anti-IL-6 clones (**D**).

Microchip-based capillary electrophoresis (LabChip 24 HT Touch; Revity) was employed to achieve more precise separation and purity assessment, offering significantly superior resolution compared to SDS-PAGE. This high-quality separation allowed for a detailed purity assessment, revealing that for anti-IL-17A clones (Figure 4A), purity ranged from 74% to 100%, with the lead clone D3 reaching 95.8% (Figure 4C). For anti-IL-6 clones (Figure 4B), purity ranged from 36% to 100%, with the lead clone A5 achieving 96.4% (Figure 4D).

**Figure 5:**
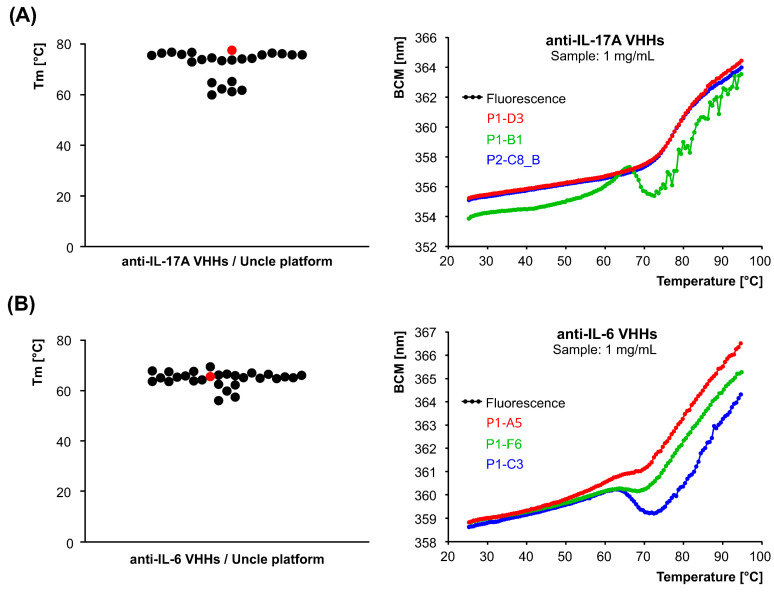
Melting temperature determination (*left panels*) for anti-IL-17A (**A**) and anti-IL-6 clones (**B**) performed using the Uncle platform, which enables the assessment of the thermal stability of the purified candidates. The most promising VHHs, D3 for IL-17A and A5 for IL-6, are marked in red. Representative melting curve analysis results for selected anti-IL-17A and anti-IL-6 clones are presented on the *right panels*.

The thermal stability of purified VHH protein samples was evaluated using the Uncle multifunctional protein stability screening platform (Unchained Labs). This analysis provided insights into the stability of the VHHs under various thermal conditions, which is critical for determining their suitability for further applications. For the anti-IL-17A clones (Figure 5A), the melting temperature ranged from 60 °C to 77.5 °C, with the lead clone D3 exhibiting a melting temperature of 76 °C. In the case of the anti-IL-6 clones (Figure 5B), the melting temperature ranged from 56 °C to 69.5 °C, with the lead clone A5 showing a melting temperature of 65.6 °C.

Combining purity assessment with thermal stability profiling allows for the selection of VHH lead candidates with optimal biophysical properties for further development. High purity ensures reliability in functional assays, while elevated thermal stability supports resilience during storage, formulation, and manufacturing, reducing the risk of instability or aggregation in later stages.

#### 3.1.3. Binding of VHHs to Human IL-17A and IL-6

**Figure 6:**
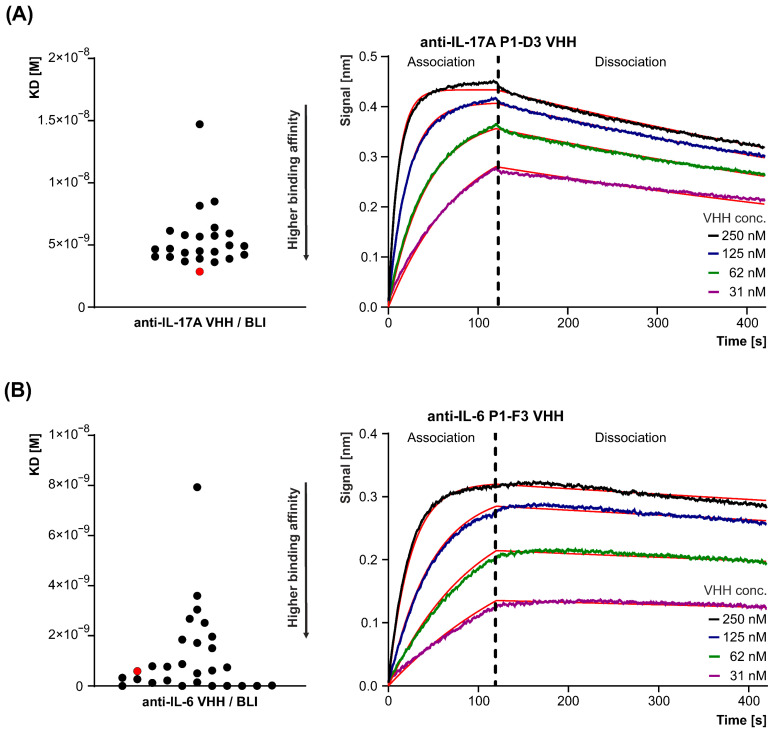
The range of KD values for VHH clones specific to anti-IL-17A (**A**) and anti-IL-6 (**B**). The most promising VHHs, D3 for IL-17A and A5 for IL-6, are marked in red on the *left panel*. On the *right panel*, representative examples of BLI data analysis are shown. The red lines indicate the fitted curves, representing the global fitting of the binding data to a 1:1 interaction model.

The binding affinity and kinetics of the purified VHHs for human IL-17A and IL-6 were evaluated using bio-layer interferometry (BLI) on the OCTET RED96 instrument (Sartorius). The equilibrium dissociation constants (KD) for both cytokines were determined using a 1:1 interaction model through global fitting. The results provided precise measurements of the binding affinities, indicating the strong and specific interactions between the VHHs and their respective cytokine targets, IL-17A and IL-6. While the KD values of the anti-IL-17A clones (Table 1, Figure 6A) ranged from 1.47 × 10^−8^ to 2.85 × 10^−9^ M, with the lead clone D3 exhibiting KD = 2.85 × 10^−9^ M, k_a_ = 3.64 × 10^5^ M^−1^s^−1^, and k_off_ = 1.04 × 10^−3^ s^−1^; the KD values for the anti-IL-6 clones ranged from 7.93 × 10^−9^ to 1.49 × 10^−12^ M, with the lead clone A5 showing KD = 5.87 × 10^−10^ M, k_a_ = 4.20 × 10^5^ M^−1^ s^−1^, and k_off_ = 2.47 × 10^−4^ s^−1^ Table 2, Figure 6B).

The low KD values, combined with favorable association (k_a_) and dissociation (k_off_) rates, highlight their strong interaction profiles, making them promising candidates for further therapeutic development.

#### 3.1.4. Assessment of VHHs’ Biological Activity Using HEK-Blue Reporter Cells

**Figure 7:**
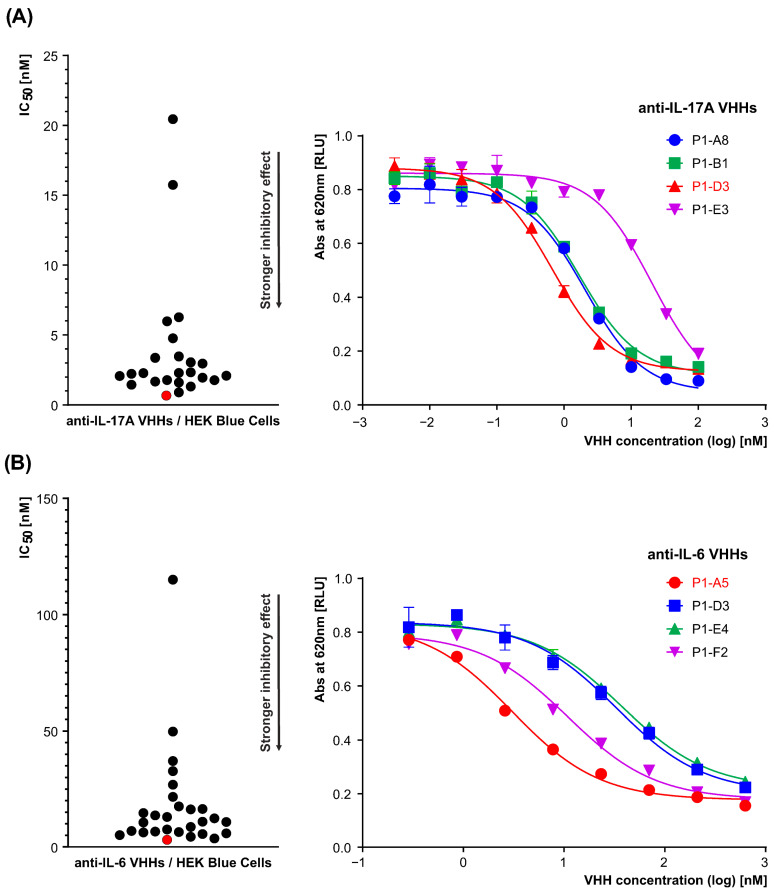
Neutralization potency of selected VHH clones targeting IL-17A (**A**) and IL-6 (**B**). The range of IC_50_ values of VHHs is shown on the *left panel*. The most promising VHHs, D3 for IL-17A and A5 for IL-6, are marked in red. *Right panel* shows representative IC_50_ determination analyses using reporter cell assays, where the dose-dependent inhibition of cytokine signaling was measured. Data were analyzed using nonlinear regression with a three-parameter dose–response model (log [inhibitor] vs. response), fitted using GraphPad Prism software. Each experiment was performed in two technical replicates.

The biological activity of the VHHs was evaluated using human IL-17A and IL-6 reporter cells (HEK293 genetic background). All selected clones demonstrated the ability to neutralize both cytokines in a dose-dependent manner. Upon IL-6 stimulation, the HEK293-derivative cells activated the STAT3-inducible *SEAP* reporter gene, leading to the release of SEAP protein into the supernatant. The VHH antibody fragments effectively inhibited this process, demonstrating their capability to neutralize IL-6 activity. Similarly, the binding of IL-17A to its receptor on HEK293 cells triggered a signaling cascade that activated NF-κB/AP-1, resulting in SEAP production. The VHHs were able to neutralize IL-17A activity, as evidenced by the reduced SEAP levels measured in the supernatant using a spectrophotometer at 620 nm. The inhibition curves were analyzed, and IC_50_ values were calculated using a three-parameter dose–response inhibition model in GraphPad Prism software. For anti-IL-17A clones, the IC_50_ values ranged from 0.67 nM to 20.45 nM, with the lead clone D3 exhibiting an IC_50_ of 0.67 nM (Table 3, Figure 7A). For anti-IL-6 clones, the IC_50_ values ranged from 3.05 nM to 115 nM, with the lead clone A5 showing an IC_50_ of 3.05 nM (Table 3, Figure 7B). For each reporter cell line, cross-reactivity controls were included to confirm the specificity of the biological activity. In the IL-17A reporter assay, the anti-IL-6 VHH clone A5 was included as a negative control and showed no biological activity, as evidenced by the absence of SEAP inhibition (Supplementary Figure S3A). Conversely, in the IL-6 reporter assay, the anti-IL-17A VHH clone D3 had no effect on IL-6-induced signaling, further confirming the target specificity of the evaluated VHHs (Supplementary Figure S3B).

Lower IC_50_ values indicate stronger target binding and more effective inhibition, providing a quantitative basis for prioritizing candidates with superior biological activity, thereby reducing the risk of selecting compounds with low biological efficacy and minimizing the risk of investing resources in candidates with limited therapeutic potential.

#### 3.1.5. Correlation Between Binding Affinity and Neutralization Potency Identifies Lead VHH Candidates

**Figure 8:**
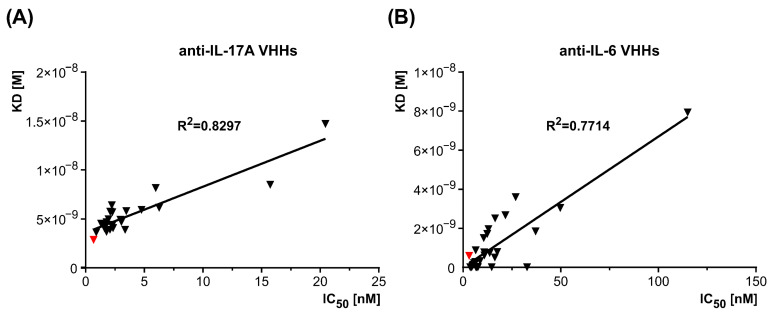
Relationship between IC_50_ values from in vitro assays and binding affinity measured by BLI. (**A**) Correlation for IL-17A, with a correlation coefficient (R^2^) of 0.8297. (**B**) Similar strong correlation for IL-6, as indicated by an R^2^ of 0.7714. The most promising VHHs, D3 for IL-17A and A5 for IL-6, are marked in red.

A comprehensive analysis was conducted to identify the most promising VHHs for each biological target, IL-17A and IL-6, based on optimal binding and neutralization parameters. Graphical comparisons between IC_50_ values obtained from in vitro assays and binding kinetics data from BLI analysis revealed strong correlations. For IL-17A (Figure 8A), the correlation coefficient (R^2^) was 0.8297, while for IL-6 (Figure 8B), it was 0.7714. These high correlation values indicate a consistent relationship between the in vitro neutralization potency and the binding affinity measured by BLI. Based on these analyses, the D3 clone was identified as the most promising candidate specific for IL-17A, exhibiting an IC_50_ value of 0.67 nM and a KD of 2.85 × 10^−9^ M. Similarly, the clone A5 was identified as the most promising candidate for IL-6, demonstrating an IC_50_ value of 3.05 nM and a KD of 5.87 × 10^−10^ M, supporting its advancement to further development. In graphical representations, these antibody fragments were marked in red to highlight their superior performance. These results underscore the potential of the VHHs to effectively neutralize key pro-inflammatory cytokines, IL-17A and IL-6, demonstrating their promise for therapeutic applications.

### 3.2. Generation and Characterization of Anti-IL17A/Anti-IL6 Bispecific Antibody CPBT0853

#### 3.2.1. Production of Antibody CPBT0853

**Figure 9:**
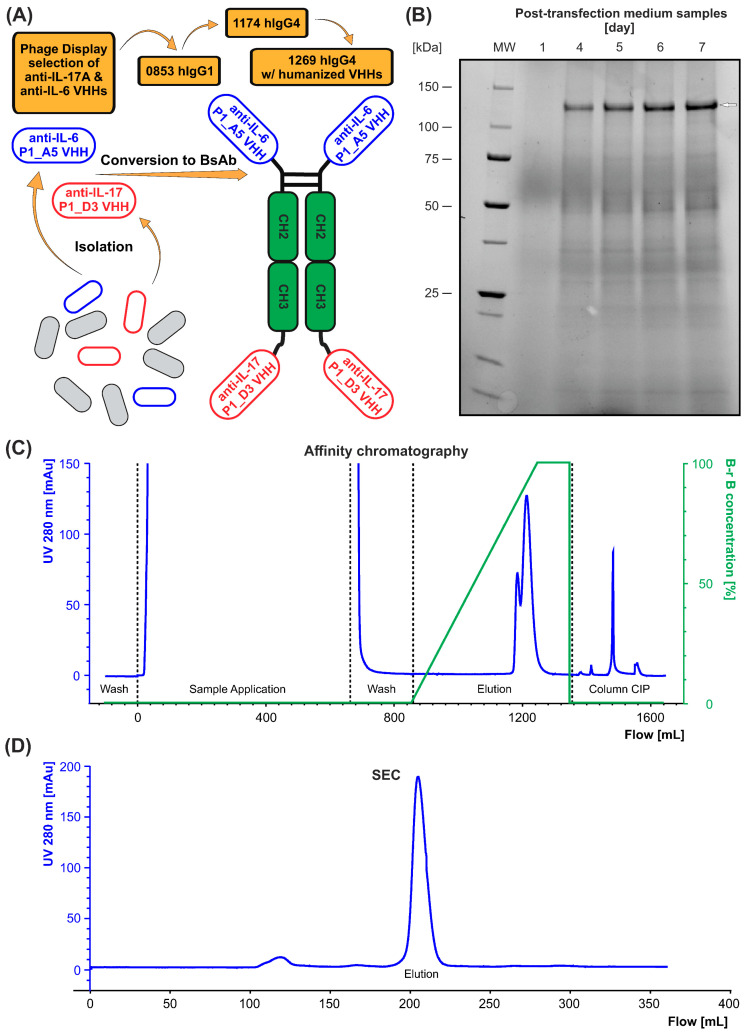
Expression and purification of CPBT0853. Panel (**A**) is a schematic representation of the generation of the bispecific antibody. Following phage display selection, and VHHs’ identification, the first produced version of bispecific antibody, named CPBT0853, was in the IgG1 format. The antibody was then reformatted into an IgG4 version (CPBT1174), and humanization was subsequently performed by substituting specific amino acids to reduce immunogenicity (CPBT1269). Panel (**B**) shows the SDS-PAGE analysis under non-reducing conditions, illustrating the expression profile of CPBT0853 over seven days following transient transfection in ExpiCHO-S cells. An arrow indicates the expected protein band. Panel (**C**) presents a representative chromatogram from the affinity purification step using MabCaptureC resin. Panel (**D**) displays the size-exclusion chromatography (SEC) profile of the final purified CPBT0853, confirming the high homogeneity of the sample.

The most promising VHHs, D3 for IL-17A and A5 for IL-6, were used as specificity-determining parts to construct synthetic single-chain bispecific antibody CPBT0853 (Figure 9A), which possesses the VHH-Fc-VHH format and was produced as a secretory protein in ExpiCHO-S cells (Figure 9B). The culture medium was collected, and the antibody was purified by affinity chromatography using MabCaptureC resin (Figure 9C). A final polishing step of the bispecific antibody was carried out by preparative size exclusion chromatography (HiLoad 16/600 Superdex 200 pg; Cytiva), where the protein resuspension buffer was exchanged to 1× PBS pH 6.8, 5% glycerol (Figure 9D). The antibody was concentrated using Amicon filters (50K MWCO; Sigma), and protein concentrations were measured by UV-VIS spectroscopy at 280 nm (NanoDrop; IMPLEN).

**Figure 10:**
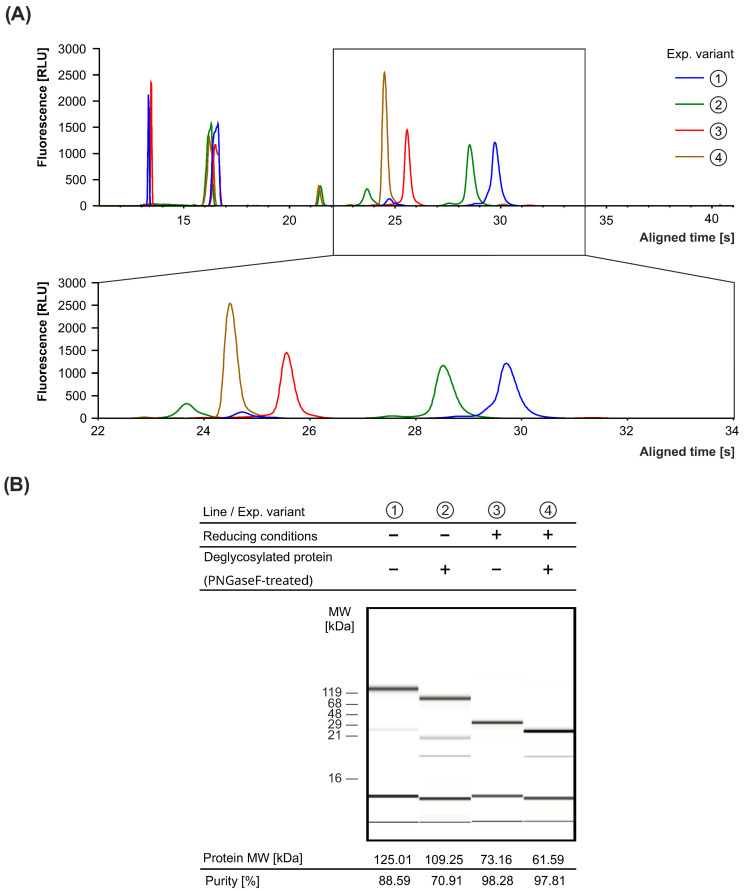
Purity analysis of CPBT0853. (**A**) Representative chromatogram obtained from capillary electrophoresis, showing the purity profile of the CPBT853 sample. (**B**) The corresponding electropherogram, providing a visual representation of the separations of the final product differentially treated, as indicated. Each subsequent line in the electropherogram (**B**) corresponds to the following peak marked in the red box (**A**).

**Figure 11:**
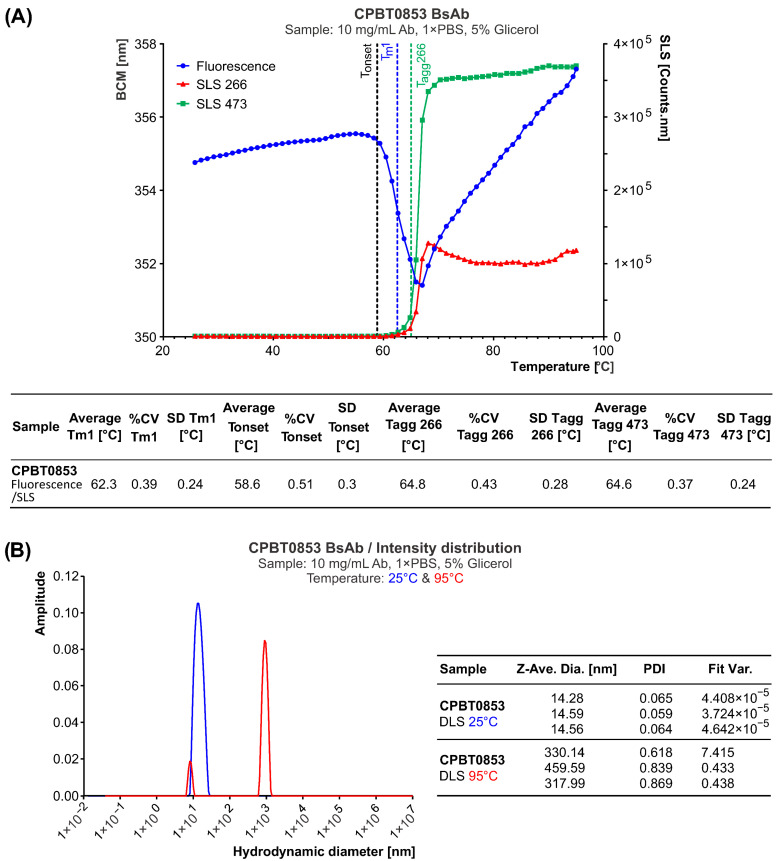
Biophysical characterization of CPBT0853 using the Uncle platform. (**A**) Thermal stability and aggregation analysis of CPBT853 assessed using fluorescence and static light scattering (SLS) to determine melting temperature (T_m_) and aggregation onset temperature (T_agg_), respectively. (**B**) Dynamic light scattering (DLS) measurements performed at 25 °C and 95 °C, demonstrating the hydrodynamic radius and polydispersity index (PDI) of the sample under native and heat-stressed conditions in three technical repetitions.

As determined by capillary electrophoresis, the resulting product exhibited high purity, approximately 98% under reducing conditions (Figure 10). Further characterization revealed a melting temperature (T_m_) of 62.3 °C, an aggregation onset temperature (T_agg_) of 64.8 °C (Figure 11A) and a polydispersity index (PDI) below 0.1 (Figure 11B). These results indicate a highly stable and homogeneous bispecific antibody preparation, suitable for further functional and structural studies.

#### 3.2.2. Binding Affinity of CPBT0853 to Human, Cynomolgus Monkey and Mouse IL-17A and IL-6

**Figure 12:**
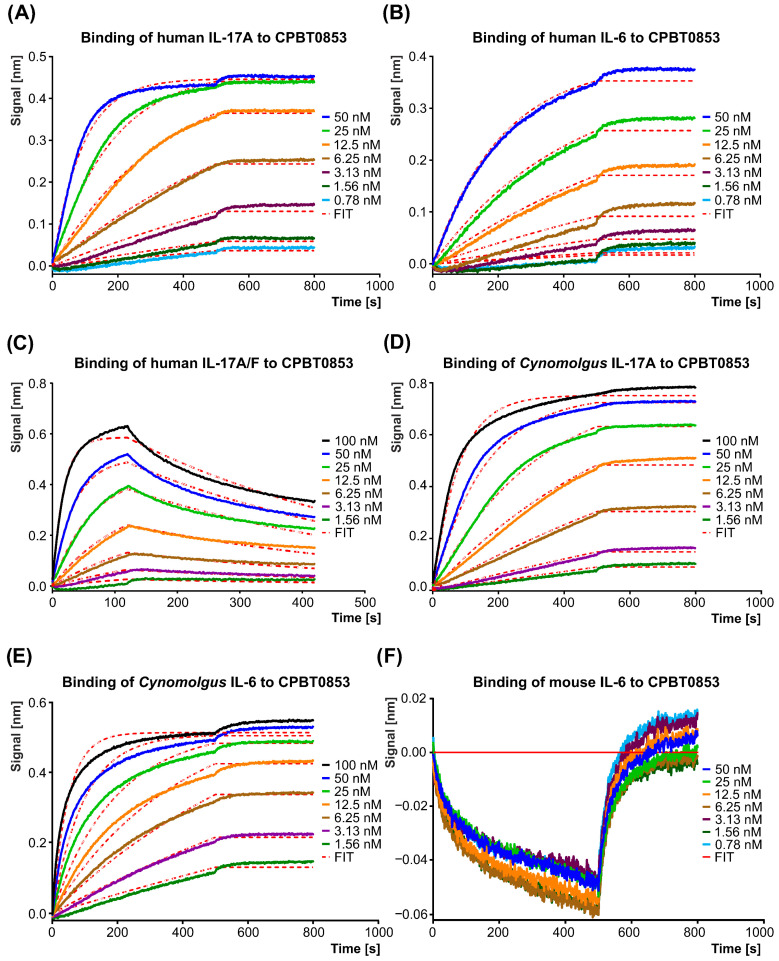
Binding affinity analysis of the CPBT0853 bispecific antibody. Representative bio-layer interferometry (BLI) binding curves for CPBT0853 interacting with human IL-17A (**A**), human IL-6 (**B**), human IL-17A/F heterodimer (**C**), cynomolgus monkey IL-17A (**D**), and cynomolgus monkey IL-6 (**E**) are shown. The absence of binding is demonstrated by the lack of interaction between CPBT0853 and mouse IL-6 (**F**).

The bispecific antibody CPBT0853, generated by formatting the single-domain antibodies, VHH A5 and D3, into an Fc format based on IgG1, was evaluated for its binding affinity and kinetics using bio-layer interferometry (BLI). CPBT0853 exhibited remarkably high affinity for both human and cynomolgus monkey IL-17A and IL-6, with equilibrium dissociation constants (KD) in the low picomolar range. The picomolar affinity observed for the Fc-fused constructs is primarily attributable to avidity effects resulting from their bivalent format, which reduces the effective dissociation rate compared to the intrinsic nanomolar affinity of monovalent VHHs. For human IL-17A (Figure 12A), a KD of 1.25 ± 0.18 pM was observed, along with an association rate constant (k_a_) of 2.40 ± 0.30 × 10^5^ M^−1^s^−1^ and a dissociation rate constant (k_off_) of 2.97 ± 0.18 × 10^−7^ s^−1^. Similarly, for human IL-6 (Figure 12B), the KD was determined to be 2.85 ± 0.33 pM, k_a_ = 1.05 ± 0.25 × 10^5^ M^−1^s^−1^ and k_off_ = 2.96 ± 0.48 × 10^−7^ s^−1^. Comparable binding parameters were observed for cynomolgus monkey cytokines, with a KD of 1.27 ± 0.24 pM for IL-17A (Figure 12D) (k_a_ = 2.34 ± 0.36 × 10^5^ M^−1^s^−1^, k_off_ = 2.91 ± 0.05 × 10^−7^ s^−1^) and a KD of 1.04 ± 0.07 pM for IL-6 (Figure 12E) (k_a_ = 3.91 ± 0.58 × 10^5^ M^−1^s^−1^, k_off_ = 3.34 ± 0.26 × 10^−7^ s^−1^). In contrast, the bispecific antibody demonstrated a significantly lower binding affinity to the human heterodimer IL-17A/F (Figure 12C), with a KD of 4.24 ± 0.47 nM (k_a_ = 4.92 ± 0.48 × 10^5^ M^−1^s^−1^, k_off_ = 2.08 ± 0.24 × 10^−3^ s^−1^). No binding was detected for mouse IL-17A and IL-6 (Figure 12F), or for human IL-17E and IL-17F homodimers, confirming the species-specific selectivity and high specificity towards IL-17A (Table 4). These results highlight the strong and specific binding capabilities of CPBT0853 to its intended targets in both human and cynomolgus monkey models, supporting further antibody development.

#### 3.2.3. Potential of CPBT0853 to Inhibit IL-17A/IL-17RA and IL-6/IL-6R Interactions

**Figure 13:**
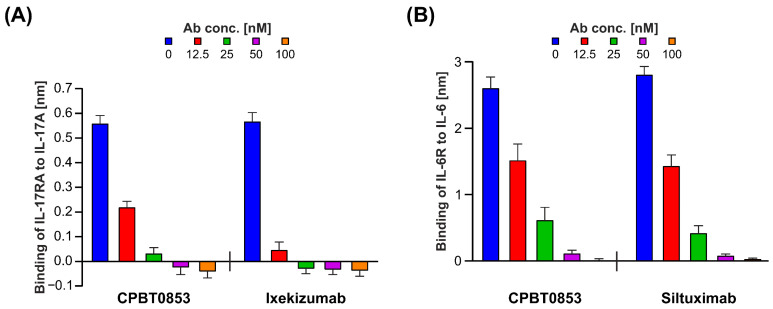
Blocking activity of the bispecific antibody CPBT0853 measured by bio-layer interferometry (BLI). Bar graphs represent quantitative data derived from BLI sensorgrams showing the effect of increasing concentrations of CPBT0853 on the binding of IL-17A (**A**) or IL-6 (**B**) to their respective receptors. The measured response [nm], reflecting the amount of receptor bound to the immobilized cytokine, decreases with increasing antibody concentration, indicating dose-dependent inhibition of cytokine–receptor interactions. Data represent mean ± SD from three independent experiments.

The potential of the bispecific antibody CPBT0853 to inhibit the interactions between human IL-17A and its receptor IL-17RA, as well as human IL-6 and its receptor IL-6R, was evaluated using bio-layer interferometry (BLI). In the assay, biotinylated IL-17A was immobilized on the BLI sensor surface. Both CPBT0853 and IL-17RA were able to bind IL-17A independently. However, when IL-17A was preincubated with CPBT0853, the subsequent binding of IL-17RA was effectively blocked in a concentration-dependent manner (Figure 13A). Representative BLI sensorgrams underlying these quantitative analyses are provided in Supplementary Figure S2. This inhibitory effect was quantified by measuring changes in binding layer thickness, and decreasing values reflected reduced receptor association. For IL-17A, CPBT0853 reduced the binding signal from 0.6 nm to 0.2 nm at a concentration of 12.5 nM, while at 50 nM, the signal dropped below zero, indicating full inhibition of receptor binding. These results are comparable to those observed for the monospecific reference antibody Ixekizumab, which produced a negative signal already at a concentration of 25 nM (Figure 13A). Similarly, CPBT0853 effectively inhibited the interaction between IL-6 and IL-6R. The binding layer thickness decreased from 2.6 nm to 1.5 nm at an antibody concentration of 12.5 nM, and negative values were observed at 100 nM, confirming complete inhibition. A similar inhibitory profile was observed for the reference antibody Siltuximab (Figure 13B). Together, these data demonstrate that CPBT0853 not only binds both IL-17A and IL-6 but also efficiently prevents their interactions with their respective receptors. The blocking potency of CPBT0853 is comparable to that of established monospecific therapeutic antibodies, suggesting the strong neutralizing capability of this bispecific molecule.

### 3.3. Evaluation of CPBT0853 in Biological Assays

#### 3.3.1. Assessment of Neutralizing Activity of Bispecific Antibody Using HEK-Blue Reporter Cells

**Figure 14:**
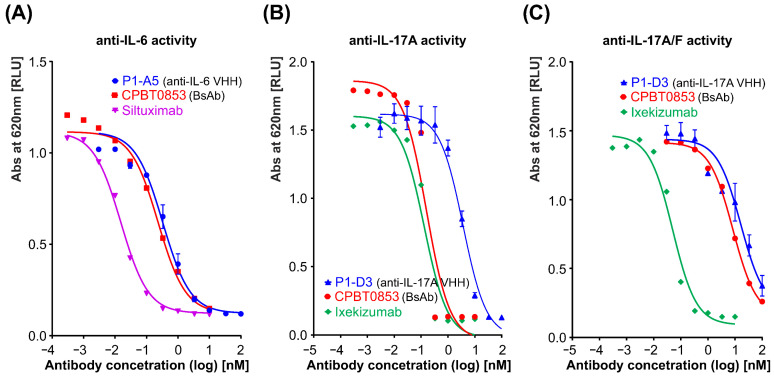
Neutralization potency of CPBT0853 targeting IL-17A (**A**) and IL-6, IL-17A. Representative graphs show IC_50_ analysis performed using a reporter cell assay. For comparison, parental VHHs and monospecific referential antibodies were used. The cellular response, measured as cytokine-induced activation, progressively declined with increasing concentrations of the tested antibodies, indicating effective inhibition of signaling through IL-6 (**A**), IL-17A (**B**), and the IL-17A/F heterodimer (**C**). Data were analyzed using nonlinear regression with a three-parameter dose–response model (log [inhibitor] vs. response), fitted using GraphPad Prism software. The curves shown represent a representative biological replicate performed with two technical replicates. The IC_50_ values summarized in Table 5 were calculated from three independent biological replicates.

The biological activity of CPBT0853 was assessed using reporter cell assays, confirming its ability to neutralize IL-17A and IL-6 signaling in a dose-dependent manner. The calculated IC_50_ values for CPBT0853 were 0.14 nM for IL-17A, 0.20 nM for IL-6, and 8.41 nM for the IL-17A/F complex, indicating potent inhibition of both cytokines, but reduced efficacy against the heterodimeric IL-17A/F complex (Table 5). For comparison, the clinically used monospecific antibodies were also evaluated. Siltuximab demonstrated greater potency than CPBT0853 in neutralizing IL-6 (Figure 14A), while Ixekizumab demonstrated comparable biological activity against IL-17A (Figure 14B) and superior efficacy in inhibiting IL-17A/F complex signaling (Figure 14C). These findings suggest that CPBT0853 may bind the IL-17A/F heterodimer with lower affinity or that steric constraints reduce its neutralization efficiency. These functional data are consistent with the bio-layer interferometry (BLI) results, confirming the high affinity and dual specificity of CPBT0853 for IL-17A and IL-6.

#### 3.3.2. Inhibition of IL-6-Induced STAT Signaling by the Bispecific Antibody

**Figure 15:**
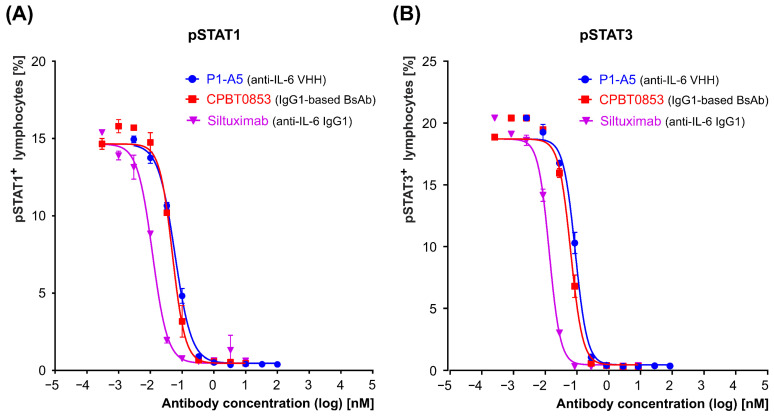
Representative IC_50_ curves showing inhibition of STATs phosphorylation in PBMCs stimulated with IL-6 and treated with increasing concentrations of the bispecific antibody CPBT0853. Phosphorylation of STAT1 (**A**) and STAT3 (**B**) was measured by flow cytometry. Data were normalized to cytokine-only controls and expressed as % pSTAT^+^ lymphocytes. IC_50_ values were calculated using a nonlinear regression with a three-parameter dose–response model (log [inhibitor] vs. response) in GraphPad Prism software. Each curve represents technical duplicates from PBMCs of one representative donor out of three tested.

To further assess the functional activity of the bispecific antibody CPBT0853, a signaling assay was performed using peripheral blood mononuclear cells (PBMCs) stimulated with IL-6. Pre-incubation of IL-6 with CPBT0853 resulted in a marked reduction in STAT1 and STAT3 phosphorylation, reflecting effective inhibition of cytokine-induced signaling (Figure 15, Table 6). The percentage of pSTAT-positive lymphocytes was quantified. The calculated IC_50_ values for CPBT0853 were 0.032 nM for pSTAT1 (Figure 15A) and 0.036 nM for pSTAT3 (Figure 15B), indicating potent blockade of IL-6-mediated pathways. For comparison, the clinically approved monospecific anti-IL-6 antibody Siltuximab demonstrated greater potency, with IC_50_ values of 0.007 and 0.008 nM for pSTAT1 and pSTAT3, respectively (Figure 15, Table 6). These results confirm that CPBT0853 neutralizes IL-6 by preventing receptor engagement and subsequent activation of the STAT pathway, albeit with slightly lower efficacy than the reference monospecific antibody.

#### 3.3.3. Evaluation of Biological Activity of Bispecific Antibodies in Human Fibroblast-like Synoviocytes (HFLS)

Human Fibroblast-Like Synoviocytes (HFLSs) were employed in this study as a disease-relevant cellular model to evaluate the anti-inflammatory activity of bispecific antibodies targeting IL-17A and IL-6. HFLS cells are key effector cells in the pathogenesis of rheumatoid arthritis, where cytokines such as IL-17A and IL-6 act directly on these cells to drive joint inflammation and damage. Therefore, HFLSs provide a physiologically relevant context to investigate therapeutic strategies aimed at neutralizing these cytokines.

To mimic the inflammatory milieu characteristic of rheumatoid arthritis, HFLSs were stimulated with IL-17A (100 ng/mL), which induced the production of pro-inflammatory cytokines, including IL-6 and IL-8. Due to the absence of mIL-6R and preserved gp130 expression, sIL-6R (100 ng/mL) was added to enable IL-6 trans-signaling to induce a pro-inflammatory response.

This stimulation elicited a pronounced increase in IL-6 secretion, rising from a basal level of 504 pg/mL to 2174.6 pg/mL. Treatment with the bispecific antibody CPBT0853 (IgG1 format), designed to simultaneously neutralize IL-17A and IL-6, resulted in a dramatic reduction in IL-6 production to 25.8 pg/mL. To delineate the relative contributions of each antigen-binding arm, CPBT0853 was converted into two monospecific control antibodies through deletion of the respective VHH domain. In comparison to CPBT0853, a monospecific anti-IL-17A antibody (CPBT1176) reduced IL-6 levels to 557 pg/mL, while a monospecific anti-IL-6 antibody (CPBT1177) lowered IL-6 to 143 pg/mL.

Despite some variability among experimental replicates—consistent with the inherent heterogeneity of primary HFLS responses—a reproducible trend toward reduced cytokine output was observed following treatment with the bispecific antibody. Furthermore, IL-8 secretion, largely driven by IL-17A, was also significantly attenuated by the bispecific antibody, with the IL-17A-targeting arm playing a predominant role in this effect.

**Figure 16:**
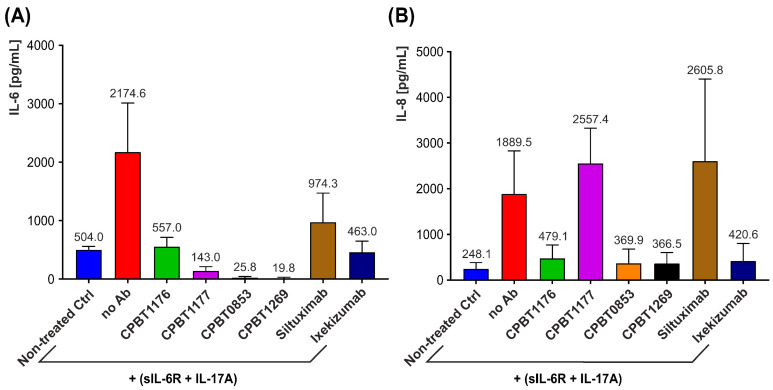
Inhibition of cytokine production (IL-6 and IL-8) in HFLS cells by CPBT0853. Levels of IL-6 (**A**) and IL-8 (**B**) secreted into the culture medium after stimulation of the cells with IL-17A. Additionally, cells were treated with bispecific and monospecific antibodies, as indicated. Data are shown as the mean ± SD from a minimum of four independent experiments. The bispecific antibody CPBT0853 effectively reduced the IL-6 and IL-8 levels, with higher efficiency, as compared to the monospecific antibodies, indicating enhanced anti-inflammatory activity. Additionally, the humanized IgG4 form of CPBT0853 (CPBT1269) was evaluated for comparison, showing activity in cytokine inhibition similar to that of the parental/original IgG1 form.

Collectively, these findings underscore the enhanced efficacy of the bispecific antibody in targeting multiple cytokine pathways simultaneously, offering a promising therapeutic approach for cytokine-driven inflammatory diseases such as rheumatoid arthritis. In addition, comparative data for a humanized IgG4 variant of CPBT0853, i.e., CPBT1269, are included to evaluate isotype-related differences in functional activity (Figure 16A,B). The IgG4 format was selected to minimize Fc-mediated effector functions, such as antibody-dependent cellular cytotoxicity (ADCC) and complement activation, relative to the IgG1 format, while preserving dual-cytokine neutralization.

## 4. Discussion

Interleukins IL-17A and IL-6 are key cytokines driving inflammatory responses and maintaining disease pathology in various autoimmune conditions. IL-17A, produced mainly by T helper17 cells, has similarly been implicated in the pathogenesis of autoimmune diseases, including rheumatoid arthritis and psoriasis [25,26]. It synergizes with other pro-inflammatory mediators, enhancing the responses of synovial fibroblasts, chondrocytes, and osteoclasts [19]. IL-6 plays a well-established role in diseases like rheumatoid arthritis by promoting the recruitment of neutrophils, stimulating B cells and T helper17 differentiation, and activating osteoclasts, contributing to joint degradation [27]. Additionally, IL-6 supports angiogenesis and induces acute-phase proteins such as CRP [28].

Conventional therapies based on monospecific antibodies, although effective in many cases, are associated with several drawbacks. These include suboptimal pharmacokinetics, systemic side effects, and insufficient control over complex and redundant inflammatory networks. Consequently, the development of multispecific antibodies has gained traction as a next-generation therapeutic strategy. These molecules offer the potential for enhanced clinical efficacy while reducing reliance on drug combinations and minimizing systemic toxicity [5,29].

The success of bispecific antibodies in oncology, e.g., Blinatumomab targeting CD20 and CD3, demonstrates their therapeutic promise in complex diseases [30]. However, bispecific formats remain largely underutilized in autoimmune disorders. To date, Ozoralizumab, which targets TNF-α and human serum albumin (HSA), is the only bispecific antibody approved for autoimmune disease (in Japan) [31]. The anti-albumin VHH primarily serves to enhance pharmacokinetic parameters, while VHHs modulate one signaling pathway. This example underscores the need for further development of a bispecific antibody modulating independent inflammatory pathways.

Experimental data suggest a strong rationale for dual targeting of IL-17A and IL-6. Ogura et al. demonstrated a feedback loop in which IL-17A amplifies inflammatory responses by inducing IL-6 expression, which, in turn, promotes further IL-17A production [20]. Although therapies with anti-IL-17A antibody like Secukinumab and Ixekizumab have shown efficacy in psoriasis and psoriatic arthritis, their benefits in rheumatoid arthritis have been limited, with clinical trials reporting outcomes comparable to those of existing approved treatments [32,33,34,35]. These results emphasize the limitations of monospecific IL-17A blockade in complex autoimmune settings and support the rationale for a multispecific approach.

In this context, our study provides evidence that a bispecific antibody targeting both IL-17A and IL-6 offers superior efficacy compared to monospecific formats. In a cellular model relevant to rheumatoid arthritis, the bispecific construct more effectively inhibited IL-6 production. Given IL-6’s central role in joint destruction and systemic inflammation, such suppression is of high therapeutic relevance [27].

Moreover, our bispecific antibody also reduced IL-8 secretion, another important chemokine regulated by IL-17A and involved in neutrophil recruitment in inflammatory diseases like rheumatoid arthritis and psoriasis [36,37]. These findings confirm that dual cytokine targeting can disrupt multiple inflammatory pathways simultaneously, offering broader disease control.

This is supported by data from Lyman et al., who reported that co-inhibition of IL-6R and IL-17A significantly reduced inflammatory cell infiltration and edema in a murine hypersensitivity model, compared to either cytokine being targeted alone [15]. Our study builds on this foundation, highlighting the translational potential of bispecific antibodies in autoimmune disease management.

Despite our promising findings, several limitations must be addressed. The current results are derived from in vitro systems, and further validation in animal models is essential to confirm efficacy and safety. Pharmacokinetic profiling, immunogenicity assessment, and long-term outcome studies will be necessary to fully characterize the clinical potential. Additionally, mechanistic studies investigating receptor occupancy, downstream signaling interference, and cytokine network disruption may further elucidate the advantages of bispecific formats.

Future research should also explore other cytokine pairs relevant to specific autoimmune contexts and investigate the optimal design parameters for bispecific antibodies to maximize target engagement and minimize off-target effects.

This study demonstrates that bispecific antibodies targeting IL-17A and IL-6 represent a promising strategy for improving outcomes in autoimmune and inflammatory diseases. By addressing multiple inflammatory pathways simultaneously, they offer the potential to overcome the limitations of current therapies and set a foundation for next-generation immunomodulatory treatments.

## 5. Patents

A patent application resulting from the work reported in this manuscript is pending: Patent Application No. P.448731.

## Figures and Tables

**Table 1:** KD values, k_a_, and k_off_, as well as R^2^ values, for all purified VHH clones anti-IL-17A. The R^2^ values reflect the accuracy of the global fitting applied during the bio-layer interferometry (BLI) analysis, ensuring the reliability of the kinetic parameters obtained. D3, the most promising VHH for IL-17A, is marked in red.

Anti-IL-17A Clones	KD [M]	KD Error	k_a_ [M^−1^s^−1^]	k_off_ [s^−1^]	R^2^
P1-A1	5.73 × 10^−9^	1.27 × 10^−11^	2.99 × 10^5^	1.71 × 10^−3^	0.9972
P1-A2	6.39 × 10^−9^	1.18 × 10^−11^	2.58 × 10^5^	1.65 × 10^−3^	0.9984
P1-A5	4.50 × 10^−9^	1.28 × 10^−11^	2.81 × 10^5^	1.27 × 10^−3^	0.9960
P1-A6	4.45 × 10^−9^	1.31 × 10^−11^	3.76 × 10^5^	1.67 × 10^−3^	0.9942
P1-A8	3.89 × 10^−9^	1.00 × 10^−11^	3.02 × 10^5^	1.17 × 10^−3^	0.9970
P1-B1	4.21 × 10^−9^	9.75 × 10^−12^	2.81 × 10^5^	1.18 × 10^−3^	0.9973
P1-B3	3.61 × 10^−9^	8.96 × 10^−12^	2.81 × 10^5^	1.02 × 10^−3^	0.9975
P1-C1	4.65 × 10^−9^	1.54 × 10^−11^	2.35 × 10^5^	1.09 × 10^−3^	0.9965
P1-C4	8.15 × 10^−9^	3.02 × 10^−11^	3.06 × 10^5^	2.50 × 10^−3^	0.9935
P1-C6	4.69 × 10^−9^	2.25 × 10^−11^	2.96 × 10^5^	1.39 × 10^−3^	0.9915
P1-C8_BIOT	4.91 × 10^−9^	1.77 × 10^−11^	3.85 × 10^5^	1.89 × 10^−3^	0.9924
P1-C11_BIOT	4.96 × 10^−9^	1.27 × 10^−11^	3.58 × 10^5^	1.78 × 10^−3^	0.9957
P1-D1	5.67 × 10^−9^	2.25 × 10^−11^	4.20 × 10^5^	2.38 × 10^−3^	0.9892
P1-D3	2.85 × 10^−9^	6.65 × 10^−12^	3.64 × 10^5^	1.04 × 10^−3^	0.9971
P1-D4	4.03 × 10^−9^	1.58 × 10^−11^	4.72 × 10^5^	1.90 × 10^−3^	0.9895
P1-E1	5.79 × 10^−9^	1.81 × 10^−11^	4.09 × 10^5^	2.37 × 10^−3^	0.9937
P1-E3	1.47 × 10^−8^	1.53 × 10^−10^	5.19 × 10^5^	7.63 × 10^−3^	0.9732
P1-E4	4.37 × 10^−9^	1.12 × 10^−11^	4.16 × 10^5^	1.82 × 10^−3^	0.9952
P1-F6	3.67 × 10^−9^	9.88 × 10^−12^	4.23 × 10^5^	1.55 × 10^−3^	0.9948
P1-G2	6.14 × 10^−9^	1.94 × 10^−11^	4.26 × 10^5^	2.62 × 10^−3^	0.9937
P1-G3	3.88 × 10^−9^	1.50 × 10^−11^	4.86 × 10^5^	1.89 × 10^−3^	0.9905
P1-G7	5.92 × 10^−9^	2.00 × 10^−11^	4.32 × 10^5^	2.56 × 10^−3^	0.9925
P1-H4	8.49 × 10^−9^	6.02 × 10^−11^	5.56 × 10^5^	4.72 × 10^−3^	0.9803
P1-H5	4.05 × 10^−9^	1.12 × 10^−11^	3.91 × 10^5^	1.58 × 10^−3^	0.9948

**Table 2:** KD values, k_a_, and k_off_, as well as R^2^ values for all purified VHH clones anti-IL-6. The R^2^ values reflect the accuracy of the global fitting applied during the bio-layer interferometry (BLI) analysis, ensuring the reliability of the kinetic parameters obtained. A5, the most promising VHH for IL-6, is marked in red.

Anti-IL-6 Clones	KD [M]	KD Error	k_a_ [M^−1^s^−1^]	k_off_ [s^−1^]	R^2^
P1-A1	2.51 × 10^−9^	2.92 × 10^−11^	2.05 × 10^5^	5.14 × 10^−4^	0.9882
P1-A2	7.93 × 10^−9^	7.47 × 10^−11^	2.09 × 10^5^	1.66 × 10^−3^	0.9767
P1-A5	5.87 × 10^−10^	1.43 × 10^−11^	4.20 × 10^5^	2.47 × 10^−4^	0.9716
P1-A6	1.96 × 10^−9^	1.78 × 10^−11^	3.33 × 10^5^	6.52 × 10^−4^	0.9820
P1-B1	5.02 × 10^−10^	2.44 × 10^−11^	1.51 × 10^5^	7.58 × 10^−5^	0.9949
P1-B2	7.67 × 10^−10^	2.01 × 10^−11^	2.25 × 10^5^	1.72 × 10^−4^	0.9894
P1-B3	7.85 × 10^−10^	2.04 × 10^−11^	1.68 × 10^5^	1.32 × 10^−4^	0.9948
P1-B4	1.49 × 10^−12^	Out of range	2.60 × 10^5^	3.86 × 10^−7^	0.9860
P1-B5	2.67 × 10^−9^	5.76 × 10^−11^	1.02 × 10^5^	2.73 × 10^−4^	0.9898
P1-B6	1.89 × 10^−11^	Out of range	3.27 × 10^4^	6.19 × 10^−7^	0.9702
P1-C3	8.70 × 10^−10^	1.31 × 10^−11^	1.70 × 10^5^	1.48 × 10^−4^	0.9979
P1-C5	1.22 × 10^−10^	1.31 × 10^−11^	1.85 × 10^5^	2.27 × 10^−5^	0.9969
P1-C6	1.43 × 10^−10^	1.31 × 10^−11^	1.82 × 10^5^	2.60 × 10^−5^	0.9972
P1-D1	3.25 × 10^−10^	1.27 × 10^−11^	2.56 × 10^5^	8.32 × 10^−5^	0.9947
P1-D2	7.37 × 10^−10^	1.70 × 10^−11^	2.03 × 10^5^	1.49 × 10^−4^	0.9947
P1-D3	6.10 × 10^−12^	Out of range	1.09 × 10^5^	6.64 × 10^−7^	0.9934
P1-D4	3.04 × 10^−9^	2.46 × 10^−11^	2.20 × 10^5^	6.68 × 10^−4^	0.9901
P1-D6	6.28 × 10^−12^	Out of range	7.11 × 10^4^	4.46 × 10^−7^	0.9867
P1-E4	1.84 × 10^−9^	9.08 × 10^−11^	2.77 × 10^4^	5.11 × 10^−5^	0.9988
P1-F2	6.12 × 10^−10^	1.09 × 10^−11^	1.77 × 10^5^	1.08 × 10^−4^	0.9987
P1-F3	1.71 × 10^−9^	1.57 × 10^−11^	1.64 × 10^5^	2.81 × 10^−4^	0.9970
P1-F4	2.49 × 10^−12^	Out of range	1.71 × 10^5^	4.24 × 10^−7^	0.9876
P1-F6_BIOT	2.07 × 10^−12^	Out of range	1.92 × 10^5^	3.97 × 10^−7^	0.9923
P1-G1	1.50 × 10^−9^	1.40 × 10^−11^	1.52 × 10^5^	2.29 × 10^−4^	0.9982
P1-G2	2.69 × 10^−10^	9.60 × 10^−12^	1.94 × 10^5^	5.22 × 10^−5^	0.9985
P1-G3	2.18 × 10^−10^	1.33 × 10^−11^	2.19 × 10^5^	4.77 × 10^−5^	0.9964
P1-G4	3.59 × 10^−9^	1.79 × 10^−11^	1.98 × 10^5^	7.12 × 10^−4^	0.9955
P1-H1	2.39 × 10^−12^	Out of range	2.35 × 10^5^	5.60 × 10^−7^	0.9867

**Table 3:** Half-maximal inhibitory concentration (IC_50_) values determined for each purified VHH clone specific to IL-6 and IL-17A. Lower IC_50_ values indicate higher neutralization capacity. The most promising VHHs, D3 for IL-17A and A5 for IL-6, are marked in red.

Anti-IL-6 Clones	IC_50_ [nM]	Anti-IL-17A Clones	IC_50_ [nM]
P1-A1	16.39	P1-A1	2.094
P1-A2	115	P1-A2	2.229
P1-A5	3.053	P1-A5	1.319
P1-A6	12.96	P1-A6	1.448
P1-B1	16.23	P1-A8	2.072
P1-B2	10.78	P1-B1	1.673
P1-B3	17.5	P1-B3	0.8989
P1-B4	7.525	P1-C1	1.783
P1-B5	21.66	P1-C4	5.973
P1-B6	6.87	P1-C6	3.045
P1-C3	6.437	P1-C8_BIOT	2.96
P1-C5	5.087	P1-C11_BIOT	1.949
P1-C6	5.861	P1-D1	2.278
P1-D1	8.647	P1-D3	0.6729
P1-D2	13.61	P1-D4	1.605
P1-D3	32.72	P1-E1	3.466
P1-D4	49.74	P1-E3	20.45
P1-D6	14.63	P1-E4	2.29
P1-E4	37.1	P1-F6	1.773
P1-F2	10.88	P1-G2	6.268
P1-F3	12.33	P1-G3	3.368
P1-F4	6.621	P1-G7	4.762
P1-F6_BIOT	4.386	P1-H4	15.74
P1-G1	10.54	P1-H5	2.335
P1-G2	6.245		
P1-G3	5.583		
P1-G4	26.89		
P1-H1	3.654		

**Table 4:** Summary of binding kinetic parameters for the bispecific antibody CPBT0853 with various cytokines including heterodimers #.

**Cytokine**	**KD [pM]**	**k_a_ [10^5^ M^−1^s^−1^]**	**k_off_ [10^−7^ s^−1^]**
Human IL-17A	1.25 ± 0.18	2.40 ± 0.30	2.97 ± 0.18
Human IL-6	2.85 ± 0.33	1.05 ± 0.25	2.96 ± 0.48
*Cynomolgus* IL-17A	1.27 ± 0.24	2.34 ± 0.36	2.91 ± 0.05
*Cynomolgus* IL-6	1.04 ± 0.07	3.91 ± 0.58	3.34 ± 0.26
**Cytokine**	**KD [nM]**	**k_a_ [10^5^ M^−1^s^−1^]**	**k_off_ [10^−3^ s^−1^]**
Human IL-17A/F	4.24 ± 0.47	4.92 ± 0.48	2 08 ± 0.24
Human IL-17E	N/B	N/B	N/B
Human IL-17F	N/B	N/B	N/B
Mouse IL-17A	N/B	N/B	N/B
Mouse IL-6	N/B	N/B	N/B

N/B—no binding detected. ^#^—values are reported as means ± standard deviations from three independent repetitions.

**Table 5:** Summary of IC_50_ values obtained from reporter cell assays assessing the neutralizing activity of CPBT0853, Siltuximab, and Ixekizumab against IL-6, IL-17A, and the IL-17A/F complex. Values represent mean IC_50_ [nM] ± standard deviation from three independent biological replicates.

IC_50_ Value [nM]			
Antibody (↓)\Target (→)	IL-6	IL-17A	IL-17A/F
VHH D3	N/A	6.67 ± 2.966	24.36 ± 7.851
VHH A5	0.42 ± 0.088	N/A	N/A
CPBT0853	0.20 ± 0.028	0.14 ± 0.027	8.41 ± 1.617
Siltuximab	0.02 ± 0.007	N/A	N/A
Ixekizumab	N/A	0.15 ± 0.014	0.07 ± 0.031

**Table 6:** IC_50_ values for inhibition of STAT1 and STAT3 phosphorylation in PBMCs stimulated with IL-6. Values represent mean ± SD from three independent biological replicates. IC_50_ values were determined using nonlinear regression with a three-parameter dose–response model (log[inhibitor] vs. response) in GraphPad Prism.

IC_50_ Value [nM]		
Antibody	pSTAT1	pSTAT3
VHH A5	0.05 ± 0.016	0.062 ± 0.037
CPBT0853	0.032 ± 0.002	0.036 ± 0.021
Siltuximab	0.007 ± 0.003	0.008 ± 0.004

## Data Availability

The original contributions presented in this study are included in this article. Further inquiries can be directed to the corresponding author.

## Supplementary Materials

The following supporting information can be downloaded at: https://www.mdpi.com/article/10.3390/antib15020029/s1, Figure S1. Workflow of VHH discovery and bispecific antibody generation targeting IL-6 and IL-17A; Figure S2. Representative BLI sensorgrams illustrating inhibition of cytokine–receptor interactions by bispecific antibody CPBT0853 and reference monospecific antibodies; Figure S3. Neutralization potency of selected VHH clones targeting IL-17A (A) and IL-6 (B).

## Abbreviations

The following abbreviations are used in this manuscript:

AHC	Anti-Human Capture
AP	Alkaline Phosphatase
BLI	Bio-Layer Interferometry
BME	β-Mercaptoethanol
BsAb	Bispecific Antibody
CD20	Cluster of Differentiation 20
CD3	Cluster of Differentiation 3
CDR	Complementarity-Determining Region
CFA	Complete Freund’s Adjuvant
CHO	Chinese Hamster Ovary cells
CRP	C-reactive protein
CXCL8	C-X-C Motif Chemokine Ligand 8/IL-8
DLS	Dynamic Light Scattering
DMARDs	Disease-Modifying Antirheumatic Drugs
EDTA	Ethylenediaminetetraacetic Acid
ELISA	Enzyme-Linked Immunosorbent Assay
Fc	Fragment Crystallizable
gp130	Glycoprotein 130 (IL-6 Signal-Transducing Receptor Subunit)
HEK	Human Embryonic Kidney
HFLS	Human Fibroblast-Like Synoviocytes
HRP	Horseradish Peroxidase
HSA	Human Serum Albumin
IC_50_	Half maximal Inhibitory Concentration
IFA	Incomplete Freund’s Adjuvant
IL-17A	Interleukin-17A
IL-17RA	Interleukin-17 Receptor A
IL-6	Interleukin-6
IL-6R	Interleukin-6 Receptor
IPTG	Isopropyl β-D-1-thiogalactopyranoside
JAK/STAT	Janus Kinase/Signal Transducer and Activator of Transcription
k_a_	Association Rate Constant
KD	Dissociation Constant
k_off_	Dissociation Rate Constant
mAb	Monoclonal Antibody
mIL-6R	Membrane-Bound IL-6 Receptor
NF-κB	Nuclear Factor Kappa-Light-Chain-Enhancer of Activated B Cells
Ni-NTA	Nickel–Nitrilotriacetic Acid
OD_600_	Optical Density at λ = 600 nm
PBMCs	Peripheral Blood Mononuclear Cells
PDI	Polydispersity Index
PNGase F	Peptide-N-Glycosidase F
pSTAT	Phosphorylated STAT (Signal Transducer and Activator of Transcription)
Ras/MAPK	Rat Sarcoma / Mitogen-Activated Protein Kinase
SEC	Size-Exclusion Chromatography
SLS	Static Light Scattering
sIL-6R	Soluble IL-6 Receptor
STAT	Signal Transducer and Activator of Transcription
T_agg_	Aggregation Temperature
T_m_	Melting Temperature
TMB	3,3′,5,5′-Tetramethylbenzidine
TNF-α	Tumor Necrosis Factor alpha
VHH	Variable Domain of Heavy-Chain-Only Antibody
