# Supplementary material for: Development of Bispecific Antibody Targeting Human IL-17A and IL-6
Source: Antibodies (Basel). 2026 Mar 30;15(2):29. doi: 10.3390/antib15020029 (PMC13113400; doi:10.3390/antib15020029)
Supplement: Supplementary file 1 [file antibodies-15-00029-s001.zip › antibodies-4142020-supplementary.pdf]

Beata Pamuła *et. al.*; Supplementary Materials

Development of bispecific antibody targeting human IL-17A and IL-6

The overall workflow leading to the generation of a bispecific antibody targeting IL-6 and IL-17A is summarized in **Figure S1**.

To obtain target-specific VHH binders, llamas were immunized with recombinant IL-6 and IL-17A antigens, resulting in the construction of a VHH phage display library with an estimated diversity of approximately 1.7×10^8 colony-forming units (cfu). Following library construction, three rounds of target-specific biopanning were performed independently for each cytokine using either biotinylated antigens captured on streptavidin-coated surfaces (denoted as biot) or antigens directly immobilized on microplate surfaces (denoted as plate), yielding 185 IL-6-derived and 183 IL-17A-derived clones.

Primary ELISA screening identified 42 IL-6-binding and 50 IL-17A-binding candidates, corresponding to 35 and 36 unique VHH clones after sequencing analysis. These candidates were further evaluated by bio-layer interferometry (BLI) off-rate screening to confirm target binding and assess dissociation kinetics, resulting in the identification of 28 IL-6 and 24 IL-17A binders with detectable binding responses.

Selected VHH clones were recombinantly expressed and purified using affinity chromatography and subjected to biophysical quality control to confirm protein integrity and purity. Their functional activity was subsequently assessed using HEK-Blue reporter cell assays measuring inhibition of IL-6- and IL-17A-mediated signalling. Based on the combined evaluation of binding and functional properties, clones A5 (anti-IL-6) and D3 (anti-IL-17A) were selected as lead candidates for further development.

These lead VHHs were subsequently combined to generate a tetravalent bispecific antibody with the architecture $(A5)_2$–Fc–$(D3)_2$, enabling simultaneous targeting of IL-6 and IL-17A. In this construct, A5 VHH domains were fused to the N-terminus and D3 VHH domains to the C-terminus of the canonical homodimeric IgG Fc.

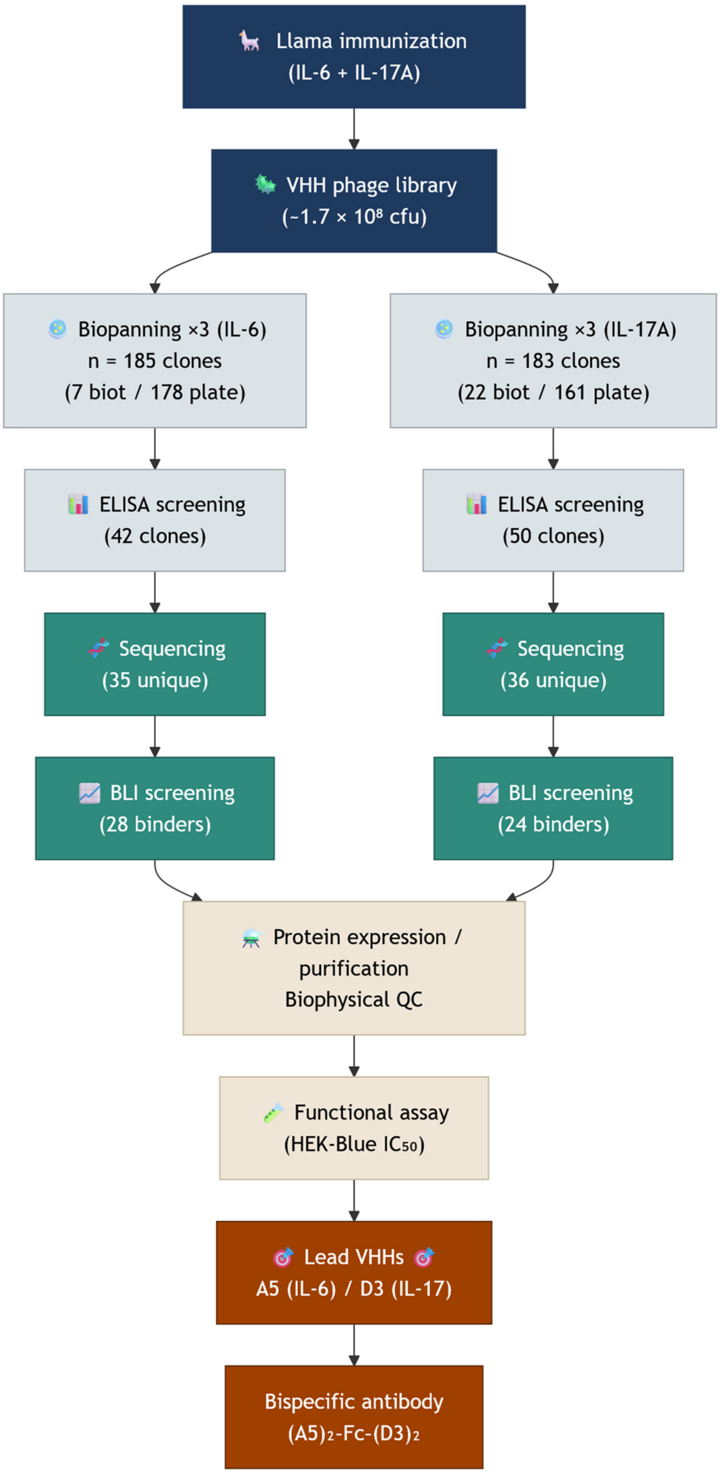

Figure S1. Workflow of VHH discovery and bispecific antibody generation targeting IL-6 and IL-17A. Llamas were immunized with IL-6 and IL-17A to generate a VHH phage display library (~1.7 × 10^8 cfu). Independent biopanning against IL-6 and IL-17A was performed using either biotinylated antigens captured on streptavidin surfaces (biot) or antigens directly immobilized on plates (plate). Binding clones were identified by ELISA, followed by sequencing and BLI off-rate screening to select unique binders. Selected VHHs were expressed recombinantly, characterized biophysically, and evaluated in HEK-Blue reporter assays. Lead clones A5 (anti-IL-6) and D3 (anti-IL-17A) were combined to generate a tetravalent bispecific antibody with the architecture $(A5)_2$–Fc–$(D3)_2$. In this figure, the notation indicates the valency of the construct, with A5 and D3 VHH domains fused to the N-terminus and C-terminus of the canonical homodimeric IgG Fc, respectively.

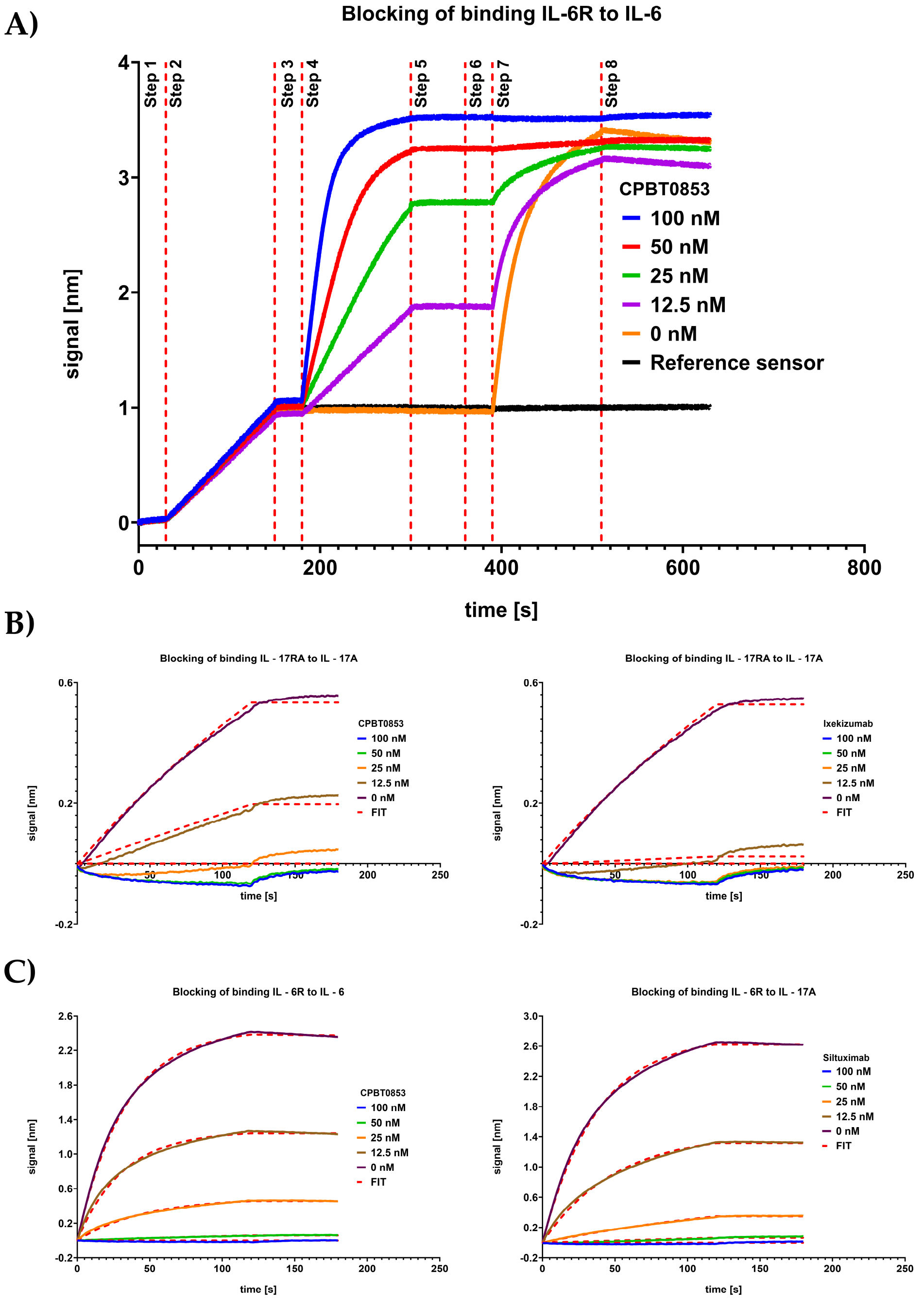

Figure S2. Representative BLI sensorgrams illustrating inhibition of cytokine–receptor interactions by bispecific antibody CPBT0853 and reference monospecific antibodies. (A) Representative sensorgram of the BLI assay workflow for blocking the interactions of IL-6 receptor with IL-6 by CPBT0853. The sequential steps are indicated as follows: (1) baseline; (2) immobilization of biotinylated IL-6 on the streptavidin sensor; (3) baseline; (4) association of CPBT0853 at increasing concentrations (12.5–100 nM); (5) dissociation; (6) baseline; (7) association of IL-6R (200 nM); (8) final dissociation. (B) Representative BLI sensorgrams showing inhibition of IL-17RA binding to IL-17A in the presence of CPBT0853 (left panel) or ixekizumab (right panel). (C) Representative BLI sensorgrams showing inhibition of IL-6R binding to IL-6 in the presence of CPBT0853 (left panel) or siltuximab (right panel). All measurements were baseline-corrected by subtraction of a reference sensor exposed only to a kinetic buffer.

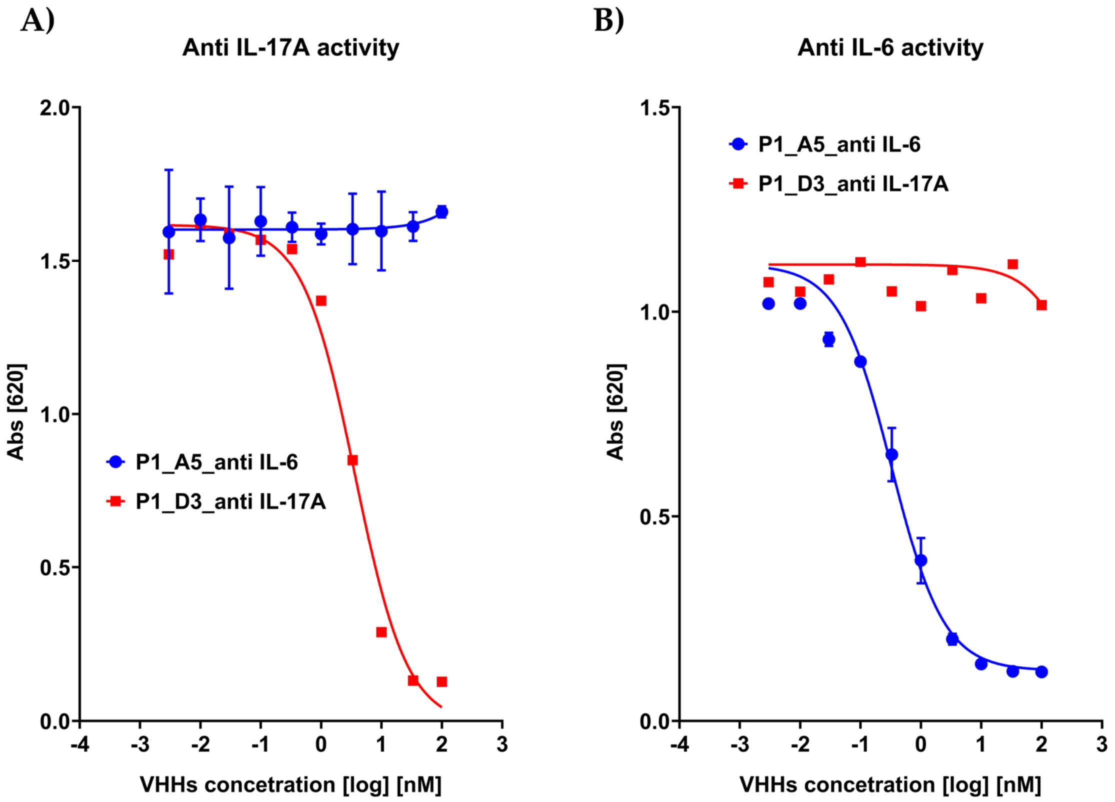

Figure S3. Neutralization potency of selected VHH clones targeting IL-17A (A) and IL-6 (B). (A) The IL-17A-specific VHH clone D3 and the IL-6-specific VHH clone A6 (negative control) were tested. D3 specifically inhibits IL-17A-induced signaling, while A6 shows no effect, confirming target specificity. (B) The IL-6-specific VHH clone A5 and the IL-17A-specific VHH clone D3 (negative control) were tested. A5 specifically inhibits IL-6-induced signaling, whereas D3 has no effect, confirming target specificity.
